# Trophic diversification and parasitic invasion as ecological niche modulators for gut microbiota of whitefish

**DOI:** 10.3389/fmicb.2023.1090899

**Published:** 2023-03-14

**Authors:** Elena N. Kashinskaya, Evgeniy P. Simonov, Larisa G. Poddubnaya, Pavel G. Vlasenko, Anastasiya V. Shokurova, Aleksey N. Parshukov, Karl B. Andree, Mikhail M. Solovyev

**Affiliations:** ^1^Institute of Systematics and Ecology of Animals, Siberian Branch of the Russian Academy of Sciences, Novosibirsk, Russia; ^2^A.N. Severtsov Institute of Ecology and Evolution of the Russian Academy of Sciences, Moscow, Russia; ^3^Papanin Institute for Biology of Inland Waters, Russian Academy of Sciences, Yaroslavl Region, Russia; ^4^Institute of Biology of the Karelian Research Centre, Russian Academy of Sciences, Petrozavodsk, Russia; ^5^Institut de Recerca i Tecnologìa Agroalimentaries (IRTA), Sant Carles de la Ràpita, Spain; ^6^Tomsk State University, Biological Institute, Tomsk, Russia

**Keywords:** Coregonidae, microbiota, 16S rRNA sequencing, tegument of cestodes, desorption, scanning and transmission electron microscopy, electron microscopy

## Abstract

**Introduction:**

The impact of parasites on gut microbiota of the host is well documented, but the role of the relationship between the parasite and the host in the formation of the microbiota is poorly understood. This study has focused on the influence that trophic behavior and resulting parasitism has on the structure of the microbiome.

**Methods:**

Using 16S amplicon sequencing and newly developed methodological approaches, we characterize the gut microbiota of the sympatric pair of whitefish *Coregonus lavaretus* complex and the associated microbiota of cestodes parasitizing their intestine. The essence of the proposed approaches is, firstly, to use the method of successive washes of the microbiota from the cestode’s surfaces to analyze the degree of bacterial association to the tegument of the parasite. Secondly, to use a method combining the sampling of intestinal content and mucosa with the washout procedure from the mucosa to understand the real structure of the fish gut microbiota.

**Results and discussion:**

Our results demonstrate that additional microbial community in the intestine are formed by the parasitic helminths that caused the restructuring of the microbiota in infected fish compared to those uninfected. Using the desorption method in Ringer’s solution, we have demonstrated that *Proteocephalus* sp. cestodes possess their own microbial community which is put together from “surface” bacteria, and bacteria which are weakly and strongly associated with the tegument, bacteria obtained after treatment of the tegument with detergent, and bacteria obtained after removal of the tegument from the cestodes.

## Introduction

In nature, in parallel with the microbiota, the digestive tract of fish is normally inhabited by different classes of helminthes (Trematoda, Cestoda, Acanthocephala, or Nematoda) characterized by different seasonal activity and impact on the host. From this perspective the fish gut could be viewed as a multi-room apartment (different parts of the gut) where each “room” is inhabited by specific lodgers (microbiota and parasites) under specific physical–chemical conditions (pH, ion and gas composition and concentration, etc.). Moreover, both types of lodgers (microbiota and parasites) are replaced by different taxonomical groups during various seasons, host ontogeny stage, or host immune status. Like all living organisms the parasites produce a number of metabolites forming the specific microenvironment that could be specified as a new ecological niche in the ecosystem of the host gut enabling colonization by specific microbiota. Indeed, it is known that cestodes may secret some organic acids (Izvekova, 2001) and, hypothetically, the pH values in the local microenvironment may be shifted to the acid side, and this can be a selective barrier for some bacterial groups. Such microenvironments permit survival on the cestode’s tegument of those bacterial species that could not colonize the gut mucosa. Such additional niches with specific microbiota that differs from microbiota of the host gut mucosa may increase the diversity of the total microbial community in the fish gut.

To date, the versatile role of parasites in different aspects of fish physiology, immunology, behavior, trophic interactions, and etc. is well documented (Bonato et al., 2018). Ignoring parasitic invasions as a factor contributing to the diversity of the microbiota may lead to biases in the interpretations of results. Since the understanding of the role of parasites in the host microbiome is relatively new, the adequate approaches to investigate such factor are poorly developed, or absent. One such methodological “blind spot” at present is the studying of gut-parasite-microbiota interactions. There is still no scientifically-based, standardized approach for collecting samples from fish gut and its parasites (Kashinskaya et al., 2017). For instance, it is unclear how to distinguish between the host microbiota and the microbial community associated with parasites as well as how to explore “true indigenous” microbiota of helminthes, which are deprived of their own digestive system. Moreover, the tegument surface has microtriches (very similar to the host’s microvilli), and this structure provides several layers where different parts of the bacterial community with various levels of adhesions may occur (Dalton et al., 2004; Poddubnaya and Izvekova, 2005; Korneva and Plotnikov, 2006). In order to study bacteria from different tegument’ layers Izvekova and Lapteva (2004) described an approach based on serial washing of bacterial cells from the tegument of cestodes *via* shaking of the whole parasite in buffers and their transfer through a series of those buffers. After that, the separate fractions of saline solution with bacterial cells were cultivated using nutrient mediums and counted without taxonomical identification. As a result of this culture-dependent approach to the study of the microbiota of cestodes, separate fractions were characterized by different levels of adhesion, but some bacterial cells could still exist in deep layers on microtriches even after many series of washings. In order to make advances toward a deeper understanding of the tapeworm’s microbiome organization an improved method is needed to separate the tegument from the cestode. The suitable approach was invented by Knowles and Oaks (1979) that, briefly, consisted of incubation of cestodes in a solution of detergent (Triton X-100) with subsequent shaking and thereafter portions fractionated by centrifugation. The combinations of these approaches with using 16S rRNA sequencing are a prospective way to create the appropriate protocol for studying the complex microbial community of cestodes infected the gut of vertebrates.

Regarding to fish gut microbiota, in many studies, the samples of mucosa and/or digesta are collected from fish gut in order to analyze the enteric microbiota. The main methodological restriction of this approach is to clean mucosa of microbial contamination from digesta and vice versa. One possible solution is to wash the intestinal segments (with mucosa attached) with saline solution. This approach was applied by Sevellec with co-authors (2018), but without robust experimental support, the outcome obtained from using this approach may be subject to bias (Solovyev et al., 2019).

Teletskoye Lake (Western Siberia) is inhabited by a sympatric pair of whitefish: small “dwarf” planktivorous form/species *Coregonus lavaretus pravdinellus* (Dulkeit, 1949) and a large “normal” benthivorous form/species *C. l. pidschian* (Gmelin, 1789) (Bochkarev and Zuikova, 2006; Bochkarev, 2009; Solovyev et al., 2022). These whitefishes are infected by mature stages of *Proteocephalus* sp. (Cestoda) in the intestine with different levels of prevalence (100% for *C. l. pravdinellus* and 45% for *C. l. pidschian*) (Bochkarev and Gafina, 1993). In the present study we have used a whitefish as a natural model of infected and uninfected fish with different feeding habits in order to gain a deeper insight into the structure of the enteric bacterial community.

The aim of the present study was to compare the composition of gut microbial communities of the sympatric pair of whitefish *C. l. pidschian* and *C. l. pravdinellus* and the associated microbiota of cestodes parasitizing their intestine using an approach described in the present study. This promising methodological approach to the study of the associated microbiota of parasites, in addition to the above mentioned protocols and methods of high-throughput sequencing can help to shed light on the relationships between parasite, fish and symbiotic microbiota.

In the present study, we have put forward several hypotheses focused on a new methodological approach and the structure of microbial communities in a host gut-parasite-microbiota system. First, we hypothesize that the primary washout procedure from the mucosa, as well as rinsing and shaking procedures for cestodes, are necessary to separate the microbiota that is weakly associated with these surfaces in order to understand in depth the real structure of the microbial communities of fish gut and cestodes. Secondarily, the gut microbiota of the studied whitefish will be affected, not only by the differences of feeding habits and other biotic and abiotic factors, but also by cestode infection. Thirdly, the microbial communities associated with fish gut and cestode will have specific taxonomic compositions. Fourthly, the associated microbiota of the parasite occupies different ecological niches within the cestode tegument and forms a parasite-specified microbial community, which on the one hand, formed a surface microbiota and, on the other hand, will form the microbiota of the deeper layer of the cestode’s tegument.

## Materials and methods

### Study area and sampling

Teletskoye Lake is a large (223 km^2^) and deep (325 m) oligotrophic lake (basin of Ob River) in the Altai Mountains (Altai Republic, Russia). In August 2019 in the north part of Teletskoye Lake (51.79°N; 87.30°E) “dwarf” *C. l. pravdinellus* (total length, TL 158.8 ± 2.6 mm, *n* = 14) infected by *Proteocephalus* sp., as well as “normal” *C. l. pidschian* uninfected (TL 252.2 ± 6.4 mm, *n* = 13) and infected by the same cestode (TL 241.3 ± 4.3 mm, *n* = 9) were collected (Supplementary Figure S1A and Supplementary Table S1). For microbiota investigations of “dwarf” whitefish we used only infected individuals due to the high prevalence level (100%) of *Proteocephalus* sp. Fish were captured using gill-nets (mesh sizes 18–25 mm) and transported alive to the laboratory in plastic containers filled with water from the site of fish capture. All fish were dissected and samples were collected aseptically. Male and female fish were identified according to gonadal development. The digestive tract (DT) was divided into three parts: stomach, anterior (without of pyloric caeca) and posterior intestine and cut separately (Supplementary Figure S1B). The content of each segment of DT were squeezed out by gentle stripping and collected separately. After collecting the content from the corresponding part of DT, the washing procedure was performed with sterile physiological saline solution (0.9% NaCl) to collect weakly adherent microbiota from mucosa of the stomach, anterior and posterior intestine of analyzed fish. Five milliliters of the solution were taken by syringe and slowly squeezed out into the “proximal” part of a vertically fixed part of the DT (stomach, anterior or posterior intestine), then when the solution passed through this part of the DT the solution was collected in an empty sterile tube at the “distal end” part of the DT. Afterward, the collected solution (washout) was stored at −80°C until analysis. After washing procedure, the upper mucosa from each segment of DT was then scraped off with a sterilized spatula and collected separately in another a sterile microcentrifuge tube.

### Desorption of associated microbiota from the tegument of cestodes

Due to high prevalence level (100%) of *Proteocephalus* sp. in “dwarf” whitefish and absent of control (uninfected) fish we used desorption method only for “normal” whitefish cestodes. Associated microbiota of *Proteocephalus* sp. were analyzed by the combinations of desorption method and method of separate the tegument from the cestode (Knowles and Oaks, 1979; Izvekova and Lapteva, 2004). The essence of the methods consists in successive washings of the microbiota from the surface of the cestode’s tegument in sterile Ringer’s solution for cold-blooded animals (pH 7.4) (Izvekova and Lapteva, 2004). The cestodes were removed immediately after dissection from nine infected fish intestine with a sterile needle or tweezers and placed in sterile Ringer’s solution to remove fragments of the host’s intestinal mucosa and content from their tegument. Depending on the size and number of worms, two to five individuals of worms were collected in one Eppendorf tube from each infected fish, the number of biological replicates from each fish were ranged from one (fish number of 3, 4, and 8) to three (fish number of 1, 2, 5, 6, 7, 9). Following this step, the Ringer’s solution fractions were frozen and also used for microbiological analysis (fraction D0). Then the first washout fraction (D1) was obtained after placing the cestodes into a new sterile Eppendorf tube with sterile Ringer’s solution and vigorously shaking on a BIOSAN TS-100 vortex for 15 s at 900 rpm. Subsequent washings D2–D5 were obtained by sequential transfer of cestodes into a new Eppendorf tube with a sterile Ringer’s solution and vigorous vortexing each for 15 min at 900 rpm. To separate the tegument from the cestodes after washing D5 the Triton X-100 detergent (0.2% w/v) was used. Subsequent washing D6 was obtained by sequential transfer of cestodes into a new Eppendorf tube with a sterile Ringer’s solution with Triton X-100 and vigorous vortexing (15 min at 900 rpm). The volume of each fraction was 1 mL. After desorption with Triton X-100 detergent, the cestodes (D7) were transferred into new tubes for isolation of the bacterial DNA. The obtained washings containing bacteria that had differential affinity to the tegument were lyophilized and used for DNA isolation (Supplementary Figure S1C). A total number of 153 samples were collected for desorption method (D0, *n* = 7; D1, *n* = 21; D2, *n* = 21; D3, *n* = 21; D4, *n* = 21; D5, *n* = 21; D6, *n* = 21; D7, *n* = 20).

### Analysis of the 28S rRNA gene of *Proteocephalus* sp. from whitefish

For genetic analysis seven individuals of *Proteocephalus* sp. from studied whitefish were collected. Total DNA was extracted using the DNA-sorb B kit manufacturers’ protocols (kit for DNA extraction, Central Research Institute of Epidemiology, Russia).

To determine the species of cestodes, partial sequences of the nuclear large subunit of rRNA gene (28S) were amplified using primers LSU5 (TAGGTCGACCCGCTGAAYTTYAGCA) and 1500R (GCTATCCTGAGGGAAACTTCG) (Littlewood et al., 2000, 2008). The cycling conditions were adopted by authors of the present study and were the following: 95°C for 5 min, 34 cycles of 95°C for 15 s, 57°C for 30 s, 72°C for 80 s; and a final extension at 72°C for 5 min. Double-stranded DNA was amplified using BioMaster HS-Taq PCR-Color (2x) kit (Novosibirsk, Russia) according to the manufacturer’s instructions.^1^

PCR reactions were 50 μL in volume and contained 25 μL BioMaster HS-Taq PCR-Color reaction mix, 10 pM of each primers, 20 μL sterile water, and 3 μL of total DNA was used as template. The PCR products were purified by adsorption on Agencourt Ampure XP (Beckman Coulter, Indianapolis, IN, United States) columns and subjected to Sanger sequencing using the BigDye Terminator V.3.1 Cycle Sequencing Kit (Applied Biosystems, Waltham, MA, United States) with subsequent unincorporated dye removal by the Sephadex G-50 gel filtration (GE Healthcare, Chicago, IL, United States). The Sanger products were analyzed on an ABI 3130XL Genetic Analyzer (Applied Biosystems). The purification and sequencing of PCR products were performed in SB RAS Genomics Core Facility (Novosibirsk, Russia). The chromatograms of the amplicon sequences were evaluated base on the sharpness and clear visibility of each peak for each nucleotide. Sites that had more than one peak for corresponding nucleotide were excluded from analysis. The sequences were manually aligned, edited and checked for unexpected stop codons in MEGA 7 (Kumar et al., 2016). Analysis of genetic distances was conducted in MEGA 7 (Kumar et al., 2016).

Sequences were deposited into GenBank (NCBI) under the following accession numbers: ON133796- ON133802.

### Scanning (SEM) and transmission (TEM) electron microscopy

To confirm and observe the effects of the desorption protocol, specimens of *Proteocephalus* sp. were sampled from “normal” whitefish using two methods. One way, immediately after dissection of the whitefish, several of the worms were removed from the fish intestine and fixed with 2.5% glutaraldehyde in 0.1 M sodium cacodylate buffer (pH 7.2). Additional specimens of *Proteocephalus* sp. were fixed with the same glutaraldehyde after desorption with 0.2% Triton X-100 detergent in order to confirm the elimination of the tegument from cestodes after this treatment. For scanning electron microscopy (SEM), after fixation in glutaraldehyde the specimens were dehydrated in a graded ethanol series, with a final change to absolute acetone. The worms were critical point-dried desiccated using a HCP-2 critical point dryer (Hitachi, Tokyo, Japan) and then mounted on stubs, sputter-coated using an JEC 1600 (Auto Fine Coater JEOL Ltd., Tokyo, Japan) with gold–palladium and examined using a JEOL JSM 6510LV scanning electron microscope (JEOL Ltd., Tokyo, Japan).

For transmission electron microscopy (TEM), after fixation in glutaraldehyde, both additional specimens from whitefish and the specimens after desorption with Triton X-100, were rinsed in 0.1 M sodium cacodylate buffer (pH 7.2) and post-fixed in 1% osmium tetroxide at 5°C for 1 h, The material was dehydrated as for the SEM and embedded in a mixture of Araldit and Epon using the instructions provided by the Araldite/Embed-812 EM Embedding Kit (EMS) (Sigma Aldrich, Buenos Aires, Argentina). Ultrathin sections (40–90 nm in thickness) were cut using a Leica MZ6 ultramicrotome (Leica Microsystems, Wetzlar, Germany), double-stained with uranyl acetate and lead citrate, and examined in a JEOL 1011 transmission electron microscope (JEOL, Ltd., Tokyo, Japan).

### Histological observation of cestodes

Intestines of infected whitefish were dehydrated in a graded series of ethanol, embedded in paraffin and cut into serial sagittal sections (3 μm thick) using a microscope Leica DMLB (Leica Microsystems, Spain). Sections were stained by Harris’ Hematoxylin and Eosin (HE) and Alcian Blue (AB) at pH 2.5 for general histomorphological observations and detection of carboxyl-rich and sulphated glycoconjugates in host mucous cells, respectively (Pearse, 1985). The histological sections were analyzed using an Olympus BX43 microscope and photographs were taken with a digital camera (Olympus UC90) with resolution of 300 dpi.

### DNA extraction, and 16S rDNA metagenomic sequencing

Before DNA extraction, all samples (mucosa, content of stomach, and intestine) were collected into sterile microcentrifuge tubes with lysis buffer (300 μL) for DNA isolation, then mechanically homogenized by pestle for 1 min. Washing of parasites and washing from mucosa of corresponding part of DT were lyophilized and used for DNA isolation. Following the kit manufacturer protocols, DNA was extracted from 100 mg of samples (excluding parasites and washings from mucosa) using a DNA-sorb B kit (Central Research Institute of Epidemiology, Russia) according to the protocol previously described (Kashinskaya et al., 2020). After extraction, the DNA concentration of all samples was determined spectrophotometrically (NanoVueTM Plus; GE Healthcare Bio-Sciences AB, Sweden), and samples were stored at −20°C for downstream processing. DNA from a sample containing only sterile deionized water was extracted and included in PCR as a negative control. Negative control was also included into amplification of the V3-V4 region of the 16S rRNA gene as other samples. No PCR bands were detected in the agarose gel.

A total number of 98, 63, and 102 samples from infected *C. l. pravdinellus*, infected and uninfected *C. l. pidschian* were collected and sequenced, respectively (Table 1).

**Table 1:** Metrics of diversity estimates of the microbial community associated with different parts of the digestive tract (DT) of whitefish and cestodes parasitizing the intestine of *C. l. pidschian*.

Fish/cestodes	Segment of DT	Type of sample	Number of analyzed samples	Number of ASV						Shannon	Simpson
				Phylum	Class	Order	Family	Genus	Total number		
Infected “dwarf” *C. l. pravdinellus*	Stomach	Content	8	11.8 ± 1.0	16.4 ± 1.6	36.8 ± 4.3	44.6 ± 5.6	41.1 ± 5.3	106.8 ± 16.8	2.7 ± 0.2	0.8 ± 0.0
		Mucosa	12	8.8 ± 1.0	11.8 ± 1.5	23.3 ± 3.3	27.8 ± 4.4	25.7 ± 4.4	55.4 ± 10.2	2.3 ± 0.3	0.7 ± 0.1
		Washout	13	10.9 ± 0.6	15.4 ± 1.0	34.0 ± 2.6	41.4 ± 3.6	42.6 ± 4.8	105.8 ± 20.6	3.3 ± 0.2	0.9 ± 0.0
		**Mean ± SE**		**10.4** ± **0.5**	**14.4** ± **0.8**	**30.9** ± **2.1**	**37.3** ± **2.8**	**36.3** ± **3.1**	**88.2 ± 10.6**	**2.8 ± 0.2**	**0.8 ± 0.0**
	Intestine (anterior)	Content	10	7.0 ± 1.0	9.7 ± 1.5	20.5 ± 3.5	23.3 ± 4.6	23.1 ± 5.3	57.5 ± 22.4	2.0 ± 0.3	0.7 ± 0.1
		Mucosa	10	5.5 ± 0.7	7.3 ± 0.8	14.0 ± 1.8	15.4 ± 2.0	16.0 ± 2.7	26.6 ± 4.3	1.3 ± 0.1	0.5 ± 0.1
		Washout	9	5.4 ± 0.6	7.0 ± 0.7	16.7 ± 1.4	17.0 ± 1.6	17.1 ± 2.0	27.7 ± 3.2	2.7 ± 0.2	0.9 ± 0.0
		**Mean ± SE**		**6.0** ± **0.5**	**8.0** ± **0.6**	**17.1** ± **1.5**	**18.6** ± **1.9**	**18.8** ± **2.1**	**37.6 ± 8.1**	**2.0 ± 0.2**	**0.7 ± 0.0**
	Intestine (posterior)	Content	12	11.4 ± 0.8	16.0 ± 1.3	35.8 ± 3.0	43.1 ± 3.8	43.6 ± 5.0	144.8 ± 2.4	2.5 ± 0.3	0.7 ± 0.1
		Mucosa	11	6.1 ± 0.6	8.5 ± 0.7	17.0 ± 1.5	20.4 ± 2.1	23.0 ± 2.8	40.9 ± 4.3	2.5 ± 0.1	0.8 ± 0.1
		Washout	13	8.2 ± 0.4	11.1 ± 0.6	24.8 ± 1.6	28.3 ± 2.0	30.6 ± 2.5	54.2 ± 3.2	3.4 ± 0.2	0.9 ± 0.0
		**Mean ± SE**		**8.6** ± **0.7**	**11.9** ± **1.2**	**26.1** ± **2.6**	**30.8** ± **3.4**	**32.6** ± **3.8**	**80.3 ± 12.9**	**2.8 ± 0.2**	**0.8 ± 0.0**
Uninfected “normal” *C. l. pidschian*	Stomach	Content	12	20.3 ± 1.6	35.2 ± 3.7	70.3 ± 6.8	89.1 ± 8.2	100.6 ± 11.1	399.0 ± 62.7	3.2 ± 0.4	0.8 ± 0.1
		Mucosa	12	8.9 ± 1.2	12.6 ± 2.4	24.3 ± 5.1	26.7 ± 5.7	26.5 ± 6.3	83.6 ± 25.5	2.8 ± 0.3	0.8 ± 0.0
		Washout	12	12.8 ± 2.0	20.8 ± 3.4	42.7 ± 7.4	55.2 ± 9.5	58.1 ± 10.5	184.5 ± 43.0	3.3 ± 0.5	0.8 ± 0.1
		**Mean ± SE**		**14.0** ± **1.3**	**22.9** ± **2.5**	**45.9** ± **5.1**	**57.1** ± **6.6**	**61.9** ± **7.9**	**223.4 ± 34.6**	**3.1 ± 0.2**	**0.8 ± 0.0**
	Intestine (anterior)	Content	13	14.6 ± 1.4	23.6 ± 3.1	51.3 ± 6.1	66.4 ± 8.0	76.5 ± 10.8	312.2 ± 59.7	4.0 ± 0.4	0.9 ± 0.1
		Mucosa	10	7.4 ± 1.1	9.0 ± 1.1	19.3 ± 2.8	23.6 ± 3.4	24.5 ± 4.2	54.6 ± 8.3	2.5 ± 0.3	0.8 ± 0.0
		Washout	10	6.2 ± 0.8	7.7 ± 1.0	14.6 ± 2.9	16.7 ± 4.0	16.0 ± 4.3	35.3 ± 10.9	1.5 ± 0.3	0.5 ± 0.1
		**Mean ± SE**		**9.9** ± **1.0**	**14.3** ± **1.9**	**30.3** ± **4.2**	**38.2** ± **5.6**	**42.2** ± **7.0**	**150.2** ± 32.8	**2.8** ± 0.3	**0.7 ± 0.0**
	Intestine (posterior)	Content	14	13.9 ± 1.2	22.3 ± 2.3	49.6 ± 5.2	63.1 ± 7.1	68.8 ± 8.5	262.2 ± 48.9	3.2 ± 0.4	0.8 ± 0.1
		Mucosa	10	6.3 ± 0.6	7.9 ± 0.8	12.9 ± 1.0	15.1 ± 0.9	14.1 ± 0.9	30.3 ± 4.5	1.8 ± 0.2	0.6 ± 0.1
		Washout	9	6.8 ± 0.6	8.8 ± 0.8	17.1 ± 1.5	18.4 ± 2.1	16.8 ± 2.5	28.3 ± 3.8	1.9 ± 0.2	0.7 ± 0.1
		**Mean ± SE**		**9.7** ± **1.0**	**14.3** ± **1.7**	**29.8** ± **3.7**	**36.6** ± **4.9**	**38.3** ± **5.7**	**128.2 ± 28.8**	**2.4 ± 0.2**	**0.7 ± 0.0**
Infected “normal” *C. l. pidschian*	Stomach	Content	8	20.7 ± 2.2	36.9 ± 5.2	74.3 ± 8.9	91.4 ± 9.9	99.5 ± 13.0	455.6 ± 108.2	3.6 ± 0.6	0.8 ± 0.1
		Mucosa	8	11.4 ± 0.9	15.6 ± 1.4	28.5 ± 2.5	33.6 ± 3.5	32.7 ± 4.9	84.3 ± 17.3	3.7 ± 0.2	0.9 ± 0.0
		Washout	7	12.9 ± 2.0	19.4 ± 3.9	37.3 ± 7.3	48.6 ± 9.2	51.2 ± 11.1	260.0 ± 71.8	3.7 ± 0.6	0.9 ± 0.1
		**Mean ± SE**		**15.1** ± **1.3**	**24.1** ± **2.8**	**47.0** ± **5.4**	**58.2** ± **6.5**	**61.5** ± **7.8**	**266.9 ± 53.4**	**3.7 ± 0.3**	**0.9 ± 0.0**
	Intestine (anterior)	Content	9	16.5 ± 0.9	27.5 ± 2.1	55.6 ± 4.9	71.1 ± 7.1	76.5 ± 8.6	370.8 ± 70.9	4.1 ± 0.6	0.9 ± 0.1
		Mucosa	9	8.2 ± 0.8	10.4 ± 1.1	19.2 ± 2.3	22.4 ± 2.9	20.4 ± 3.2	37.8 ± 7.1	2.3 ± 0.3	0.7 ± 0.1
		Washout	4	6.6 ± 1.4	7.8 ± 1.5	11.6 ± 2.8	13.4 ± 3.9	11.6 ± 4.9	15.75 ± 4.3	1.87 ± 0.3	0.74 ± 0.1
		**Mean ± SE**		**11.3** ± **1.0**	**16.9** ± **2.0**	**32.6** ± **4.4**	**40.6** ± **5.9**	**41.6** ± **6.8**	**170.0 ± 46.1**	**2.9 ± 0.3**	**0.8 ± 0.0**
	Intestine (posterior)	Content	8	16.6 ± 2.0	29.8 ± 4.3	64.2 ± 8.8	80.0 ± 11.9	92.0 ± 16.4	602.6 ± 124.9	5.1 ± 0.3	0.9 ± 0.0
		Mucosa	4	9.6 ± 2.1	13.6 ± 4.2	25.7 ± 8.5	30.1 ± 10.6	31.7 ± 13.0	60.5 ± 38.6	2.0 ± 0.4	0.6 ± 0.1
		Washout	6	7.7 ± 0.6	10.5 ± 0.9	21.4 ± 2.0	24.6 ± 2.3	25.2 ± 2.6	53.0 ± 15.8	2.2 ± 0.5	0.7 ± 0.1
		**Mean ± SE**		**11.7** ± **1.3**	**19.1** ± **2.8**	**38.3** ± **6.2**	**46.7** ± **8.0**	**51.0** ± **10.2**	**298.9 ± 85.3**	**3.4 ± 0.4**	**0.8 ± 0.1**
Cestodes		D0	**7**	11.4 ± 2.0	18.4 ± 4.2	37.7 ± 8.5	47.4 ± 11.0	52.3 ± 13.6	218.1 ± 91.6	2.8 ± 0.6	0.7 ± 0.1
		D1	21	5.2 ± 0.4	6.6 ± 0.5	14.7 ± 1.1	19.2 ± 1.2	21.7 ± 1.5	38.7 ± 2.7	1.9 ± 0.2	0.7 ± 0.0
		D2	21	4.9 ± 0.5	6.4 ± 0.6	13.3 ± 1.2	17.0 ± 1.6	18.2 ± 2.0	34.2 ± 4.6	1.5 ± 0.2	0.6 ± 0.1
		D3	21	4.7 ± 0.2	6.0 ± 0.2	12.6 ± 0.7	15.5 ± 1.0	15.6 ± 1.2	29.1 ± 2.5	1.7 ± 0.2	0.6 ± 0.1
		D4	21	4.0 ± 0.3	5.2 ± 0.3	11.8 ± 0.8	14.7 ± 0.9	15.7 ± 1.2	27.9 ± 2.1	1.7 ± 0.2	0.6 ± 0.1
		D5	21	4.3 ± 0.2	5.7 ± 0.2	11.7 ± 0.6	14.6 ± 0.9	15.8 ± 1.2	28.6 ± 2.4	1.7 ± 0.2	0.7 ± 0.1
		**Mean ± SE**		**4.6** ± **0.2**	**6.0** ± **0.2**	**12.8** ± **0.4**	**16.2** ± **0.5**	**17.4** ± **0.7**	**31.7 ± 1.4**	**1.7 ± 0.1**	**0.6 ± 0.0**
		D6	21	4.2 ± 0.2	5.6 ± 0.2	10.8 ± 0.7	13.9 ± 0.9	15.1 ± 1.1	26.5 ± 2.1	1.7 ± 0.2	0.6 ± 0.1
		D7	20	5.6 ± 0.2	7.4 ± 0.3	16.5 ± 0.8	22.5 ± 1.0	28.2 ± 1.4	51.1 ± 2.3	2.2 ± 0.2	0.7 ± 0.0

The bold character indicates mean and standard error.

### 16S rDNA metagenomic sequencing

Sequencing of the V3, V4 hypervariable regions of 16S rRNA genes was carried out on an Illumina MiSeq sequencing platform (600 cycles – 2 × 300 paired-end) by Evrogen (Moscow, Russia) using the primer pair S-D-Bact-0341-b-S-17, 5′-CCTACGGGNGGCWGCAG-3′ and S-D-Bact-0785-a-A-21, 5′-GACTACHVGGGTATCTAATCC-3′ (Klindworth et al., 2013).

The amplification conditions and other methods were applied according to the original manufacturer’s protocol.^2^ The PCR reaction contained at least 2.5 μL of DNA (5 ng μL^−1^), 5 μL of reverse primer (1 μM), 5 μL of forward primer (1 μM) and 12.5 μL of 2× KAPA HiFi Hotstart ReadyMix (KAPA Biosystems, Wilmington, MA, United States) in a total volume of 25 μL. The PCR reaction was performed on a 96-well 0.2 mL PCR plate (Life Technologies) using the following program: 95°C for 3 min, followed by 25 cycles of 95°C for 30 s, 55°C for 30 s and 72°C for 30 s and a final extension step at 72°C for 5 min. After producing amplicons, the libraries were cleaned up using AMPure XP beads (Beckman Coulter). Samples were multiplexed using a dual-index approach with the Nextera XT Index kit (Illumina Inc., San Diego, CA, United States) according to the manufacturer’s instructions. Raw sequence data were deposited in the Sequence Read Archive (SRA NCBI) under accession number PRJNA814856.

### 16S sequence processing

Quality control of obtained reads was done with FastQC v. 0.11.9 and MultiQC v. 1.13 software (Andrews, 2010; Ewels et al., 2016). The Cutadapt v. 4.1 (Martin, 2011) was used to trim primer sequences from both forward and reverse reads with the primer finding and removal step repeated two times. We used DADA2 v. 1.24 pipeline (Callahan et al., 2016) to process raw 16S reads into amplicon sequence variants (ASVs) at the 100% nucleotide identity. Forward reads were trimmed by 15 bp on the 5′ end and truncated at position 250, while reverse reads were truncated at position 200. We also discarded any reads contained more than two expected errors. The minimum number of total bases to use for error rate learning (‘learnErrors’ function) was set to 109 with randomization allowed. Then the reads were dereplicated, denoised and merged with default parameters. Resulting ASVs shorter that 350 bp were discarded, and chimeric sequences were removed with ‘removeBimeraDenovo’ function.

The IDTAXA algorithm (Murali et al., 2018) of the DECIPHER v. 2.24 R package (Wright, 2016) was used to assign taxonomy to each ASV with training set SILVA SSU r138 (modified).^3^ We retained for further analysis only ASVs assigned to Bacteria at least at phylum level. All singleton and doubleton ASVs were filtered from samples. The final dataset consisted of 14,907 ASVs and 416 samples. Mean number of reads across individuals was 17,790 (range 1,007–83,327) (details in the Supplementary Text S1). Read count data at the different taxonomical levels were shown in the Supplementary Table S2.

### Alpha diversity and taxonomic composition

The number of observed ASVs and diversity estimates (Shannon and Simpson indexes) per sample were calculated using phyloseq v. 1.40 package for R (McMurdie and Holmes, 2013). This package was also used to summarize abundance by taxonomic ranks. MS Excel 2019 was used to create bar chart graphs and tables with mean and standard error (mean ± SE). For estimating the diversity differences between groups, a non-parametric Kruskal-Wallis test with Dunn’s multiple comparisons test was applied as implemented in R. The same tests were also used to compare the differential abundance of dominant ASVs between different types of samples in control and infected fish using PAST v. 3.16 (Hammer et al., 2011).

### Beta diversity

For the analysis of beta diversity the dataset was cleaned by prevalence, removing any ASVs appearing only in one sample. This resulted in a dataset with 4,978 ASVs and mean number of reads across individuals 17,054 (range 828–81,639). Read counts were transformed to proportions, without rarefication. A Bray–Curtis dissimilarity matrix was calculated in phyloseq and used for downstream analyses. Permutational multivariate analysis of variance using distance matrices (PERMANOVA) was used as implemented in the ‘adonis2’ function of the vegan v. 2.6.4 R package (Oksanen et al., 2018). To control for the individual variability when testing hypotheses, the individual fish was used as a blocking factor to constrain permutations. Multiple pairwise comparisons for all pairs of levels of used factors were performed using the ‘pairwise.adonis2’ function of pairwiseAdonis v. 0.4 R package (Martinez Arbizu, 2020). The FDR correction of pairwiseAdonis results was performed with our custom script.^4^ Analysis of multivariate homogeneity of group dispersions (variances) to test if one or more groups is more variable than the others, was performed using the ‘betadisper’ function of the vegan. In all the aforementioned tests statistical significance was determined by 10,000 permutations. To visualize differences among groups of samples we performed principal coordinates analysis (PCoA) in vegan package.

The Linear discriminant analysis effect size (LEfSe) algorithm (Segata et al., 2011) hosted on a Galaxy web application^5^ was used to identify biomarkers that were significantly different in relative abundance among the analyzed groups. The alpha value for the factorial Kruskal-Wallis test among classes was set to 0.01 and the threshold on the logarithmic LDA score for discriminative features was set to 4.0 with the one-against-all strategy for multi-class analysis.

## Results

### 28S analysis, general histological view of *Proteocephalus* sp. and electron microscopy (SEM and TEM) description of its proglottid tegument before and after desorption with Triton X-100 detergent

A 1,452 bp fragment of the 28S rRNA gene, was amplifed and sequenced from seven specimens of *Proteocephalus* sp.; all sequences from studied cestodes were identical. Hence, we consider cestodes from studied whitefishes belong to be the same species of genus *Proteocephalus*.

**Figure 1:**
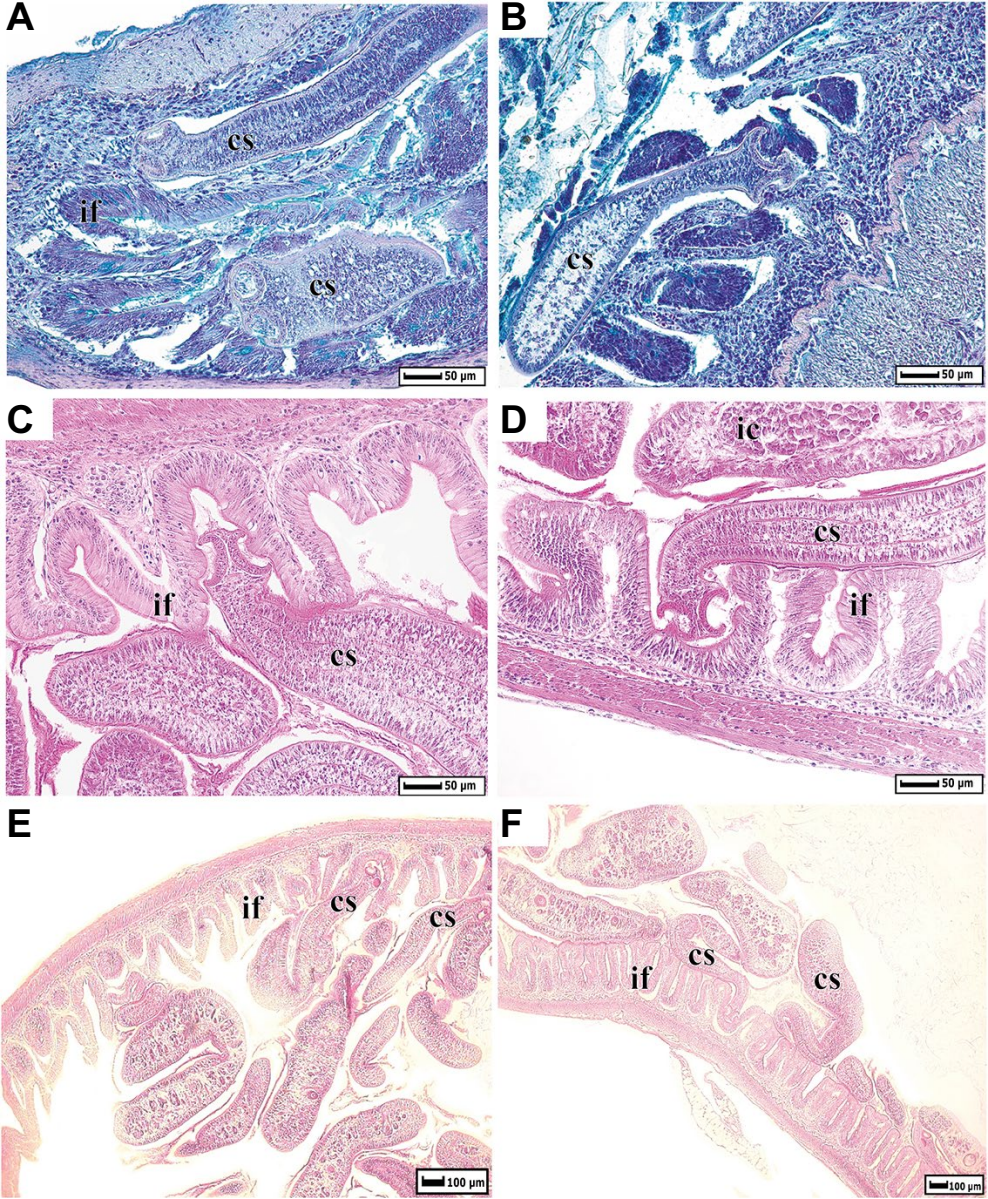
Histological dissections of whitefish intestine infected by *Proteocephalus* sp. **(A,B)** – sections were stained by Alcian Blue at pH 2.5; **(C–F)** by Harris’ Hematoxylin and Eosin. cs, cestode; if, intestinal folds; ic, intestinal content.

The histological sections of intestine of whitefish infected by *Proteocephalus* sp. were shown in Figure 1. Several tapeworms are attached by their scoleces to the folds of intestine with the strobilae lying within the intestinal lumen, other cestodes simply lying in the intestinal lumen.

**Figure 2:**
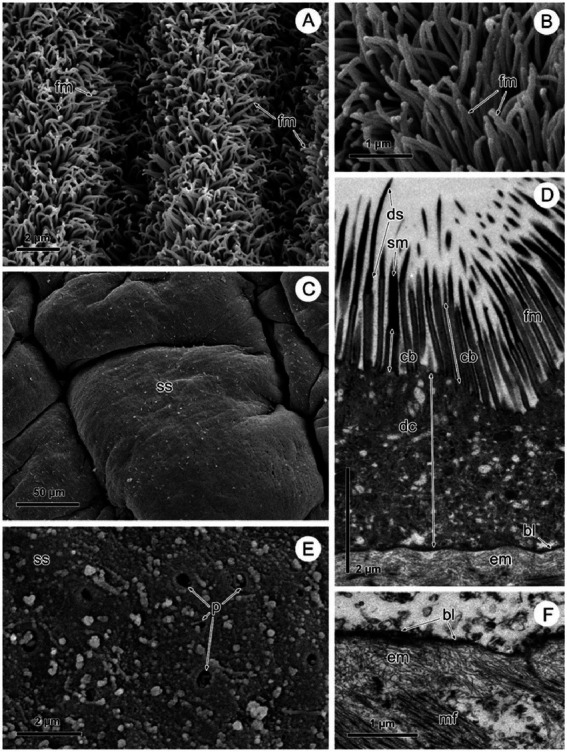
SEM and TEM observation of *Proteocephalus* sp. surface before **(A,B,D)** and after **(C,E,F)** Triton X-100 desorption. **(A,B)** SEM view of arrangement of filamentous microtriches on strobila surface. **(C)** SEM view of smooth surface of strobila. **(D)** TEM view of the tegument showing distal syncytial cytoplasm covered with microtriches and supported by basal lamina and fibrillar extracellular layer. **(E)** SEM view of the surface losing distal syncytial cytoplasm, note places of the connection of distal cytoplasm with sunken perikarya. **(F)** TEM of a portion of the tegument losing distal cytoplasm with microtriches, note basal lamina with fibrillar extracellular layer along the border of the tapeworm. bl, basal lamina; cb, cylindrical base of microtriches; dc, distal syncytial cytoplasm; ds, distal shaft of microtiches; em, extracellular matrix; fm, filamentous microtriches; mf, muscle fibers; p, pores; sm, spiniform microtriches; ss, smooth surface.

By SEM, there was the typical highly dense arrangement of long, remarkably flexible filamentous microtriches revealed throughout the strobila in naturally infected *Proteocephalus* sp. (Figures 2A,B). The TEM images of these specimens shows that the tegument is composed of an external anucleate cytoplasmic layer (distal syncytial cytoplasm) covered with numerous long slender filamentous microtriches interspersed with individual spiniform microtriches (Figure 2D). Filamentous microtriches with a long, cylindrical base and shorter electron-dense pointed distal shaft are considered to increase the absorption area and thus facilitate uptake of nutrients. Spiniform microtriches have a shorter and wider cylindrical base and longer and wider electron-dense distal shaft. The distal tegumental cytoplasm lies on the basal lamina consisting of the outer dense homogeneous layer and the inner fibrillar extracellular layer (Figure 2D). By SEM, the surface of *Proteocephalus* sp. after desorption with Triton X-100 detergent was smooth (Figure 2C) and devoid of surface microtriches (Figure 2E). Visible pores on the surface of the body are the places of the connection of sunken tegumental perikarya with distal syncytial cytoplasm *via* cytoplasmic processes (Figure 2E). Shapeless, small fragments of residue or fragments of tegumental composition are visible on the surface (Figure 2E). TEM investigation of experimental specimens of *Proteocephalus* sp. distinguished only the occurrence of electron-dense basal lamina on the surface of these tapeworms (Figure 2F).

### Alpha-diversity of microbial community associated with different parts of the digestive tract of whitefishes

#### “normal” whitefish

In general, alpha-diversity of microbial community associated with infected “normal” whitefish digestive tract was reduced from highest to lowest, as follows: stomach content- > intestinal content - > washout from stomach - > stomach mucosa - > intestinal mucosa - > washout from intestine.

##### Stomach

In infected “normal” whitefish the highest richness and diversity estimates were observed in samples of the stomach content (ASV: 455.6 ± 108.2, Shannon: 3.6 ± 0.6, Simpson: 0.8 ± 0.1, correspondingly), while the lowest value was detected in the stomach mucosa (ASV: 84.3 ± 17.3, Shannon: 3.7 ± 0.2, Simpson: 0.9 ± 0.0) (Table 1). In uninfected “normal” whitefish similar tendencies of the values of indices were observed: the highest ASV, Shannon, and Simpson indexes were registered in stomach content (399.0 ± 62.7, 3.2 ± 0.4, and 0.8 ± 0.1, correspondingly) and the lowest one in mucosa (83.6 ± 25.5, 2.8 ± 0.3, and 0.8 ± 0.0, correspondingly). According to the Dunn’s test there were no significant differences in the diversity estimates of the stomach microbial community between uninfected and infected “normal” whitefish (Dunn’s test, *p* > 0.05). Significant differences in the number of ASV were found between the microbiota associated with the mucosa and content of stomach of “normal” whitefish (Dunn’s test, *p* > 0.05).

##### Intestine

In infected “normal” whitefish the highest richness and diversity estimates (ASV, Shannon, Simpson) were observed in content of the posterior intestine (602.6 ± 124.9, 5.1 ± 0.3, and 0.9 ± 0.0, correspondingly), while the lowest one was detected in the mucosa of anterior intestine (37.8 ± 7.1, 2.3 ± 0.3, 0.7 ± 0.1, correspondingly) (Table 1). In uninfected “normal” whitefish the highest richness and diversity estimates (ASV, Shannon, Simpson) were observed in content of the anterior intestine (312.2 ± 59.7, 4.0 ± 0.0, and 0.9 ± 0.1, correspondingly), while the lowest one was detected in the washout from the mucosa of posterior intestine (28.3 ± 3.8, 1.9 ± 0.2, 0.7 ± 0.1, correspondingly). The infection status of fish (infected, uninfected) in all cases had no significant determinative effect on the composition of the microbiota (Dunn’s test, *p* > 0.05). The segment of the digestive tract analyzed (anterior or posterior intestine) also had no significant determinative effect on the composition of the microbiota (Dunn’s test, *p* > 0.05). But, the significant differences in the number of ASV were found between the microbiota associated with the mucosa and content of anterior and posterior intestine of both uninfected and infected “normal” whitefish (Dunn’s test, *p* < 0.05).

#### “dwarf” whitefish

In general, alpha-diversity of microbial community associated with infected “dwarf” whitefish digestive tract was reduced from highest to lowest, as follows: stomach content- > washout from stomach - > intestinal content - > stomach mucosa - > washout from intestine - > intestinal mucosa.

##### Stomach

The highest richness and diversity estimates were observed in samples of the stomach content and washout of stomach mucosa (ASV: 106.8 ± 16.8 and 105.8 ± 20.6, Shannon: 2.7 ± 0.2 and 3.3 ± 0.2, Simpson: 0.8 ± 0.0 and 0.9 ± 0.0, correspondingly), while the lowest value was detected in the mucosa of infected fish (ASV: 55.4 ± 10.2, Shannon: 2.3 ± 0.3, Simpson: 0.7 ± 0.1) (Table 1). There are no significant differences in the alpha-diversity estimates of microbial community between the stomach mucosa and content of infected “dwarf” whitefish (Dunn’s test, *p* > 0.05).

##### Intestine

The highest richness and diversity estimates (ASV: 144.8 ± 22.4, Shannon = 2.5 ± 0.3, Simpson = 0.7 ± 0.1) were observed in content of posterior intestine, while the lowest ones were detected in the mucosa of anterior intestine of infected “dwarf” whitefish (ASV: 26.6 ± 4.3, Shannon: 1.3 ± 0.1, Simpson: 0.5 ± 0.1) (Table 1). The segment of the intestine (anterior or posterior intestine) and type of sample (mucosa or content) analyzed in several cases had a significant determinative effect on the composition of the microbiota of infected “dwarf” whitefish (Supplementary Table S3). Thus, significant differences in number of ASV were found in mucosa when comparing the microbiota associated with the anterior and posterior intestinal content of “dwarf” whitefish (Dunn’s test, *p* < 0.05). The number of ASV were also significantly different between mucosa and content of posterior intestine of “dwarf” whitefish (Supplementary Table S3).

##### Between forms of whitefishes

Significant differences in the diversity estimates (the number of ASV, Shannon, Simpson) were found for anterior content and mucosa when comparing the microbiota of “dwarf” and uninfected “normal” whitefish, whereas comparisons of microbiota among “dwarf” and infected “normal” whitefish were shown the significant differences only for content of anterior and posterior intestines (Dunn’s test, *p* < 0.05) (Supplementary Table S3).

### Alpha-diversity of microbial community associated with washout from the stomach and intestinal mucosa of whitefish

#### “normal” whitefishes

There were no significant differences in the diversity estimates of the microbial community between the washout from the mucosa of anterior and posterior intestine in comparison with their intestinal mucosa of the corresponding part of DT of infected “normal” whitefish (Dunn’s test, *p* > 0.05). In uninfected “normal” whitefish the microbiota of the washout from the stomach mucosa and mucosa of the anterior intestine was significantly different from their mucosa only for number of ASV (Dunn’s test, *p* < 0.05).

#### “dwarf” whitefish

When comparing Shannon and Simpson values among washout samples from mucosa of the anterior intestine and their intestinal mucosa of infected “dwarf” whitefish, significant differences were found (Dunn’s test, *p* < 0.05). The significant differences were also found for number of ASV between washout from stomach mucosa and their mucosa of infected “dwarf” whitefish (Supplementary Table S3).

### Alpha-diversity of microbial community associated with cestodes parasitizing the intestine of “normal” whitefish

Alpha-diversity of the microbial community associated with different fractions of cestodes obtained before and after desorption are shown in Table 2. According to Dunn’s test (at *p* < 0.05) the significantly highest richness (ASV: 218.1 ± 91.6) and diversity estimates (Shannon: 2.8 ± 0.6, Simpson: 0.7 ± 0.1) were observed in the D0 fraction in comparison with D1-D6 fractions. The number of ASV in the microbiota of the D7 fraction (55.1 ± 2.3) was also significantly different (Dunn’s test, *p* < 0.05) than in the D2-D6 fractions (from 26.5 to 34.2) (Supplementary Table S3).

**Table 2:** Alpha- and beta-diversity of microbial community of different washout fractions of cestodes obtained before and after desorption.

Comparison			Alpha-diversity						Beta-diversity	
			Richness estimates		Diversity estimates				ADONIS	
			ASV		Shannon		Simpson			
			Z statistic	Adjusted *p*-value	Z statistic	Adjusted *p*-value	Z statistic	Adjusted *p*-value	R2	*P*-value corrected
D0	vs.	D1	2.34	**0.022**	1.61	0.122	0.86	0.352	0.06	**0.003**
		D2	3.08	**0.003**	2.40	**0.042**	1.58	0.259	0.07	**0.005**
		D3	3.37	**0.001**	1.95	0.093	0.95	0.410	0.07	**0.002**
		D4	3.55	**0.001**	1.99	0.094	0.99	0.416	0.07	**0.002**
		D5	3.50	**0.001**	1.85	0.097	0.91	0.364	0.07	**0.002**
		D6	3.67	**0.001**	1.91	0.091	0.83	0.319	0.17	**0.002**
D1	vs.	D2	1.04	0.213	1.13	0.234	1.02	0.465	0.01	**0.003**
		D3	1.46	0.118	0.48	0.421	0.13	0.538	0.01	0.062
		D4	1.72	0.082	0.54	0.408	0.18	0.550	0.01	**0.040**
		D5	1.65	0.089	0.34	0.454	0.07	0.515	0.02	0.062
		D6	1.88	0.060	0.43	0.427	−0.04	0.483	0.11	**0.002**
D2	vs.	D3	0.42	0.406	−0.65	0.388	−0.88	0.357	0.00	0.603
		D4	0.67	0.335	−0.59	0.401	−0.83	0.331	0.01	0.072
		D5	0.60	0.351	−0.78	0.371	−0.94	0.388	0.01	0.060
		D6	0.84	0.279	−0.69	0.382	−1.06	0.523	0.10	**0.002**
D3	vs.	D4	0.25	0.464	0.06	0.504	0.05	0.493	0.00	0.891
		D5	0.19	0.465	−0.14	0.518	−0.06	0.504	0.00	0.930
		D6	0.42	0.419	−0.04	0.496	−0.17	0.535	0.10	**0.002**
D4	vs.	D5	−0.07	0.473	−0.20	0.507	−0.11	0.513	0.00	0.930
		D6	0.16	0.460	−0.11	0.515	−0.22	0.548	0.09	**0.002**
D5	vs.	D6	0.23	0.459	0.09	0.506	−0.11	0.528	0.10	**0.002**
D7	vs.	D0	−1.21	0.170	−0.75	0.370	−0.24	0.560	0.07	0.060
		D1	1.57	0.100	1.19	0.222	0.86	0.335	0.09	**0.002**
		D2	2.60	**0.011**	2.30	**0.048**	1.86	0.161	0.12	**0.002**
		D3	3.01	**0.003**	1.66	0.124	0.99	0.448	0.13	**0.002**
		D4	3.26	**0.002**	1.72	0.118	1.04	0.490	0.12	**0.002**
		D5	3.20	**0.002**	1.53	0.134	0.93	0.374	0.13	**0.002**
		D6	3.43	**0.001**	1.62	0.127	0.82	0.311	0.20	**0.002**

The bold character indicates significance at *p* < 0.05.

### Beta-diversity of associated microbiota of different segments of digestive tract of whitefish

#### “normal” whitefish

The type of sample (mucosa, content) and the segment of the DT (stomach, anterior and posterior intestine) had a significant determinative effect on the composition of the “normal” whitefish microbiota. Thus, the differences in associated microbiota of the “normal” whitefish were significant between mucosa and content for all segments of the DT (ADONIS test, Bray-Curtis matrix, *p* < 0.05). But, when comparing the microbiota among different segments of the DT, significant differences were not found. Significant differences in the beta-diversity were also found for anterior content and mucosa when comparing the microbiota of “normal” whitefish stomach and intestine (Supplementary Table S3).

**Figure 3:**
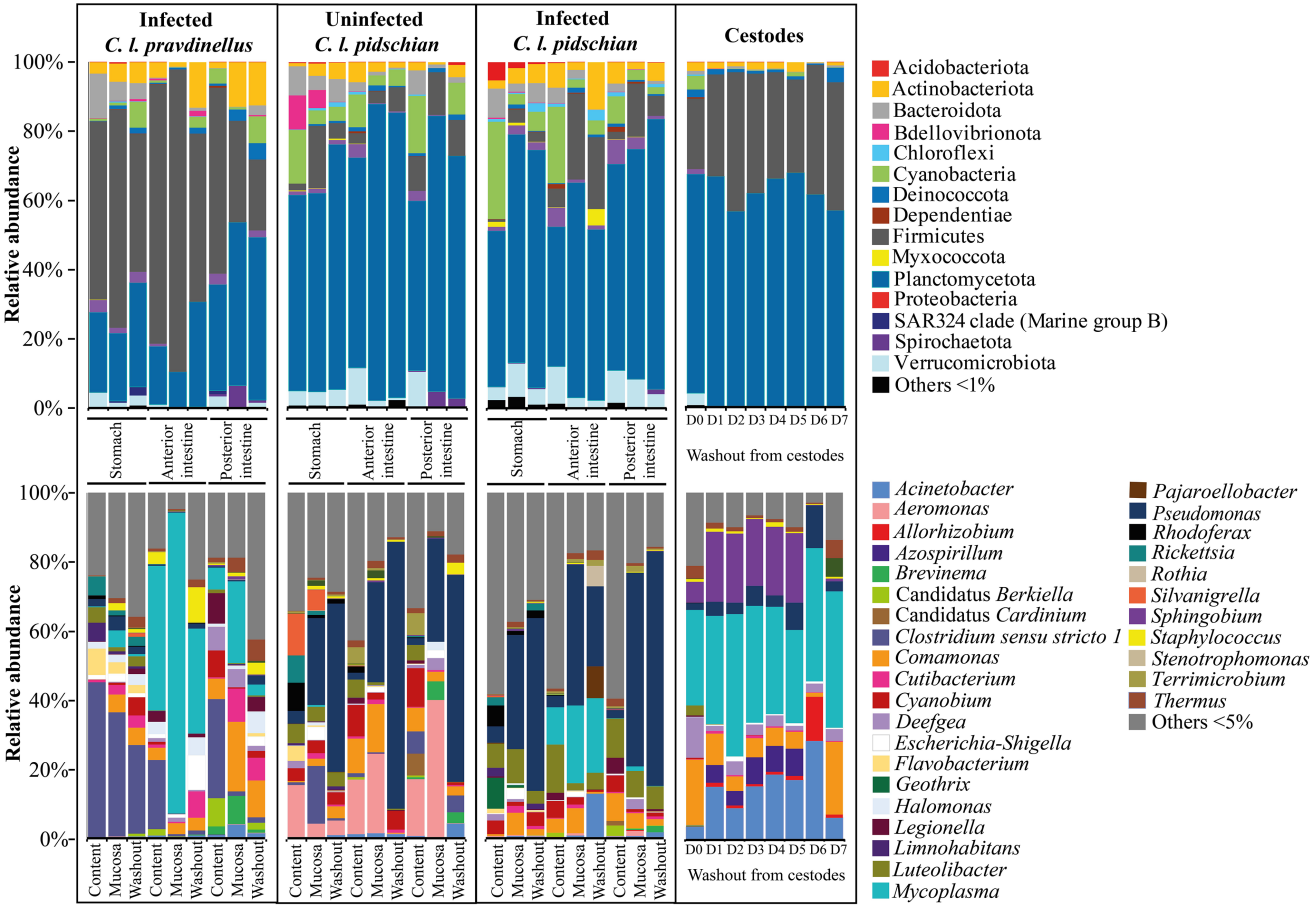
Dominant ASV at the phylum and lowest taxonomical level within the microbial communities from different segments of the digestive tract of whitefish and microbiota associated with cestodes parasitizing the intestine of “normal” *C. l. pidschian*.

Thirty-nine phyla were registered in the microbiota associated with the stomach and intestine of the infected and uninfected “normal” whitefish. The dominant microbiota of the stomach mucosa and content from the infected “normal” whitefish was represented by Proteobacteria (65.9 and 45.1%, correspondingly), Verrucomicrobiota (9.6 and 3.7%, correspondingly), Actinobacteriota (4.5 and 2.3%, correspondingly), Cyanobacteria (3.1 and 28.2%, correspondingly), Bacteroidota (2.4 and 8.4%, correspondingly), and Acidobacteriota (1.7 and 5.4%, correspondingly) (Figure 3). In the anterior and posterior intestine (mucosa and content) of the infected “normal” whitefish, the relative abundance of Proteobacteria remained dominant and occupied from 40.3 to 66.5% of the total composition of phyla. Other dominant phyla in the anterior and posterior intestine (mucosa and content) of the infected “normal” whitefish were represented by Firmicutes (2.1–24.8%), Cyanobacteria (2.3–22.0%), Actinobacteriota (2.0–7.1%), Verrucomicrobiota (2.8–10.5%), and Planctomycetota (0.9–7.1%). In uninfected “normal” whitefish the dominant phyla were similarly represented in stomach and intestine with the exception of the phylum Bdellovibrionota, in which relative abundances was low in comparison with infected fish.

The dominant ASV at the lowest taxonomical level (Figure 3) in microbiota of the stomach mucosa and content of infected “normal” whitefish were *Pseudomonas* (33.0 ± 4.1 and 4.7 ± 3.5%, correspondingly), *Luteolibacter* (9.8 ± 2.7 and 6.9 ± 1.7%, correspondingly), *Comamonas* (6.4 ± 0.8 and 0.5 ± 0.2%, correspondingly), *Geothrix* (0.8 ± 0.6 and 9.0 ± 6.0%, correspondingly), and *Rhodoferax* (1.2 ± 0.8 and 6.1 ± 2.7%, correspondingly), whereas, the dominant microbiota of mucosa and content from the stomach of uninfected “normal” whitefish were represented by *Pseudomonas* (25.0 ± 7.0 and 3.8 ± 3.1%, correspondingly), *Clostridium sensu stricto 1* (16.7 ± 7.4 and 0.8 ± 0.8%, correspondingly), *Aeromonas* (4.0 ± 2.2 and 15.7 ± 8.4%, correspondingly), *Silvanigrella* (6.0 ± 6.0 and 12.1 ± 8.0%, correspondingly), and *Rhodoferax* (0.9 ± 0.5 and 8.2 ± 3.7%, correspondingly). Included among the dominant microbial community of the mucosa and content of the anterior and posterior intestine of infected “normal” whitefish were *Pseudomonas* (from 2.3 to 56.0%), *Comamonas* (from 4.1 to 8.0%), *Luteolibacter* (from 2.1 to 13.9%), *Mycoplasma* (from 1.3 to 22.5%). Whereas, the microbiota of the mucosa and content of the anterior and posterior intestine of uninfected fish were *Pseudomonas* (from 1.9 to 29.8%), *Aeromonas* (from 15.8 to 39.5%), *Comamonas* (from 2.7 to 13.9%), and *Cyanobium* (from 2.0 to 11.1%). According to the ADONIS test on Bray-Curtis matrix significant differences were obtained for stomach mucosa and anterior and posterior content between infected and uninfected fish (*p* < 0.05) (Supplementary Table S2).

**Figure 4:**
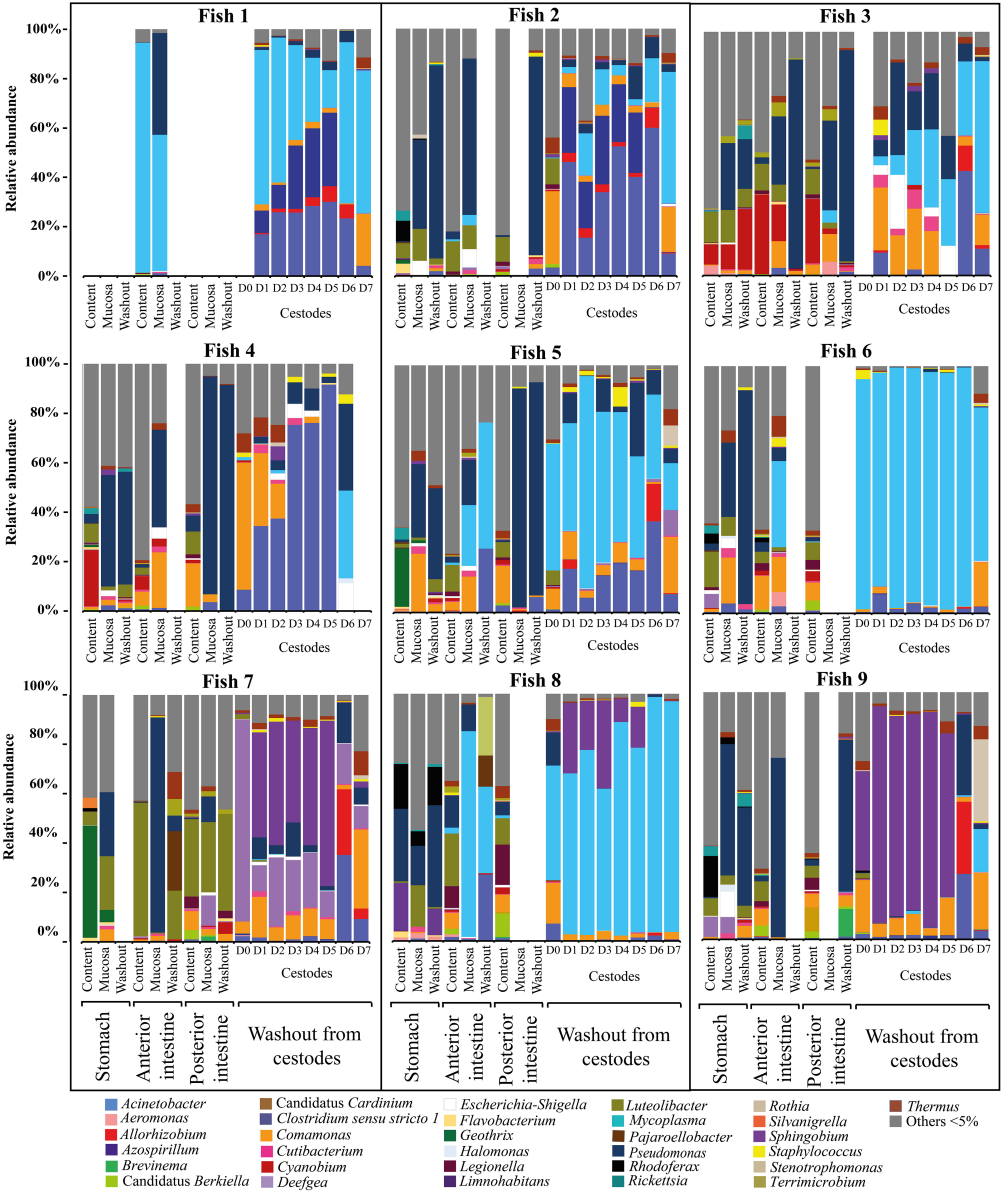
Individual microbial community of infected “normal” *C. l. pidschian* and microbiota associated with cestodes parasitizing the intestine of fish.

The factor «forms/species» had also a significant determinative effect on the composition of the microbiota of “normal” whitefish (Supplementary Table S3). As shown on Figure 4 the relative abundance of dominant bacteria of infected “normal” whitefish were varied depending on each individual of fish.

#### “dwarf” whitefish

The type of sample (mucosa, content) had a significant determinative effect on the composition of the “dwarf” whitefish microbiota (Supplementary Table S3). The differences in associated microbiota of the “dwarf” whitefish were significant between mucosa and content of the anterior and posterior intestine (ADONIS test, Bray-Curtis matrix, *p* < 0.05). When comparing the microbiota of stomach and different segments of the intestine (anterior and posterior), the differences were also significant.

Twenty-nine phyla were registered in the microbiota associated with the stomach and intestine of the “dwarf” whitefish, with Firmicutes (up to 87.0%) presenting as the dominant phylum in the stomach, anterior and posterior intestine (Figure 3). At the lowest taxonomical level, the microbiota associated with the stomach mucosa and content of infected “dwarf” whitefish were mainly represented by *Clostridium sensu stricto 1* (35.8 ± 12.0 and 45.0 ± 11.9%, correspondingly). The microbiota of mucosa and content of anterior intestine were dominated by *Mycoplasma* (87.0 ± 3.4 and 41.7 ± 10.6%, correspondingly), and *Clostridium sensu stricto 1* (0.8 ± 0.5 and 19.9 ± 11.1%, correspondingly); whereas, the microbiota of mucosa and content of the posterior intestine were dominated by *Clostridium sensu stricto 1* (1.3 ± 0.7 and 28.6 ± 9.7%, correspondingly), *Comamonas* (20.0 ± 4.3 and 5.9 ± 1.6%, correspondingly), *Cutibacterium* (9.6 ± 7.6 and 0.5 ± 0.1%, correspondingly), and *Mycoplasma* (23.7 ± 7.0 and 6.3 ± 3.5%, correspondingly).

##### Between forms/species of whitefish

The relative abundances of the dominant phyla and ASV at the lowest taxonomical level in both whitefish varied depending on the different segment of the DT (stomach, anterior and posterior intestine) and the type of sample (content, mucosa, or washout from the mucosa). Significant differences in microbiota associated with mucosa and content of all segments of the DT between infected “dwarf” whitefish, and infected and uninfected “normal” whitefish were obtained (ADONIS test, Bray-Curtis matrix, *p* < 0.05) (Supplementary Table S3). It is interesting to note that for the anterior intestine of both of the infected whitefish, the ratio of the *Mycoplasma* in the samples of the mucosa and content changed in a similar way in comparison with uninfected “normal” whitefish. The highest abundance of this genus registered in the mucosa of the anterior rather than in the posterior intestines, and stomach mucosa and content.

### Beta-diversity of associated microbiota of washout fractions from the stomach and intestinal mucosa of whitefish

According to the ADONIS test based on Bray-Curtis matrix (Supplementary Table S3) there were no significant differences between the microbiota of the washings from the intestinal mucosa from both anterior and posterior intestines of infected “normal” whitefish. In contrast to the infected “normal” whitefish, the microbiota of the mucosa of the anterior intestine from uninfected “normal” whitefish were significantly different in comparison with their washings (*p* < 0.05). The significant differences were also observed between the mucosa and their washing from the posterior intestine of infected “dwarf” whitefish (*p* < 0.05).

### Desorption of associated microbiota from the tegument of cestodes parasitizing the intestine of “normal” whitefish and their relationship to the host

The type of fraction had a significant determinative effect on the composition of the microbiota associated with cestodes. Thus, the fraction D0, D6 and D7 were significantly different in comparison with all others fractions (*p* < 0.05) in exception with fraction D0 and D7 when comparing it between each other’s. Significant differences were also found between fraction D1 and fraction D2 and D4 (*p* < 0.05). In other cases, there were no significant differences when comparing the fraction D1, D2, D3, D4, and D5 between each other’s (*p* > 0.05).

Twenty-nine phyla were registered in the microbiota associated with the cestodes parasitizing the intestine of “normal” whitefish. At the phylum level the dominant microbiota of all fractions were mainly represented by Proteobacteria (from 56.8 to 68.0%) and Firmicutes (from 20.4 to 40.2%) (Figure 3). The relative abundances of these phyla did not significantly differ between various fractions from the tegument of cestodes (Dunn’s test, *p* > 0.05). At the lowest taxonomical level, in the cestode’s fraction D0 the dominant bacteria were represented by *Comamonas* (19.0 ± 6.6%), *Deefgea* (11.8 ± 11.7%), and *Mycoplasma* (27.5 ± 13.9%), and *Sphingobium* (6.1 ± 8.9%). The microbiota of fraction D1-D5 were dominated by *Acinetobacter* (15.0 ± 1.8%), *Mycoplasma* (32.8 ± 1.7%), and *Sphingobium* (19.9 ± 0.9%). The dominant position of these bacteria remained stable in fraction D6 with the exception of *Sphingobium* (0.2 ± 0.06%), which was replaced by more abundant *Allorhizobium* (12.7 ± 2.7%) and *Pseudomonas* (12.4 ± 2.7%). The microbiota associated with fraction D7 were represented by *Acinetobacter* (6.2 ± 1.0%), *Comamonas* (21.0 ± 3.2%), and *Mycoplasma* (39.3 ± 7.6%). Relative abundance of main dominants associated with different fractions were shown in Supplementary Figure S2.

**Figure 5:**
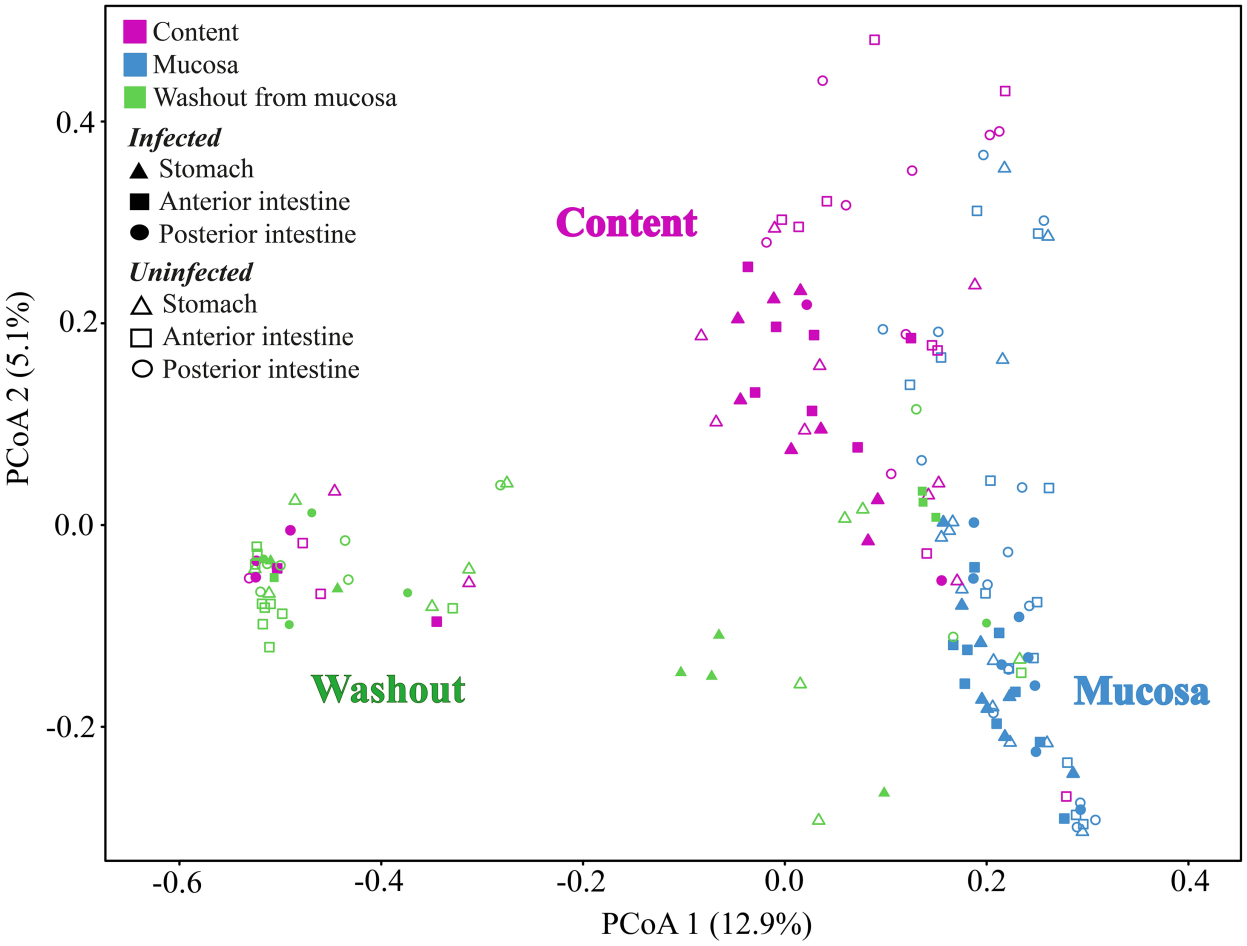
Principal coordinates analysis (PCoA) for microbial communities of different segments of the digestive tract of uninfected and infected “normal” *C. l. pidschian* and cestodes parasitizing the intestine of fish.

**Figure 6:**
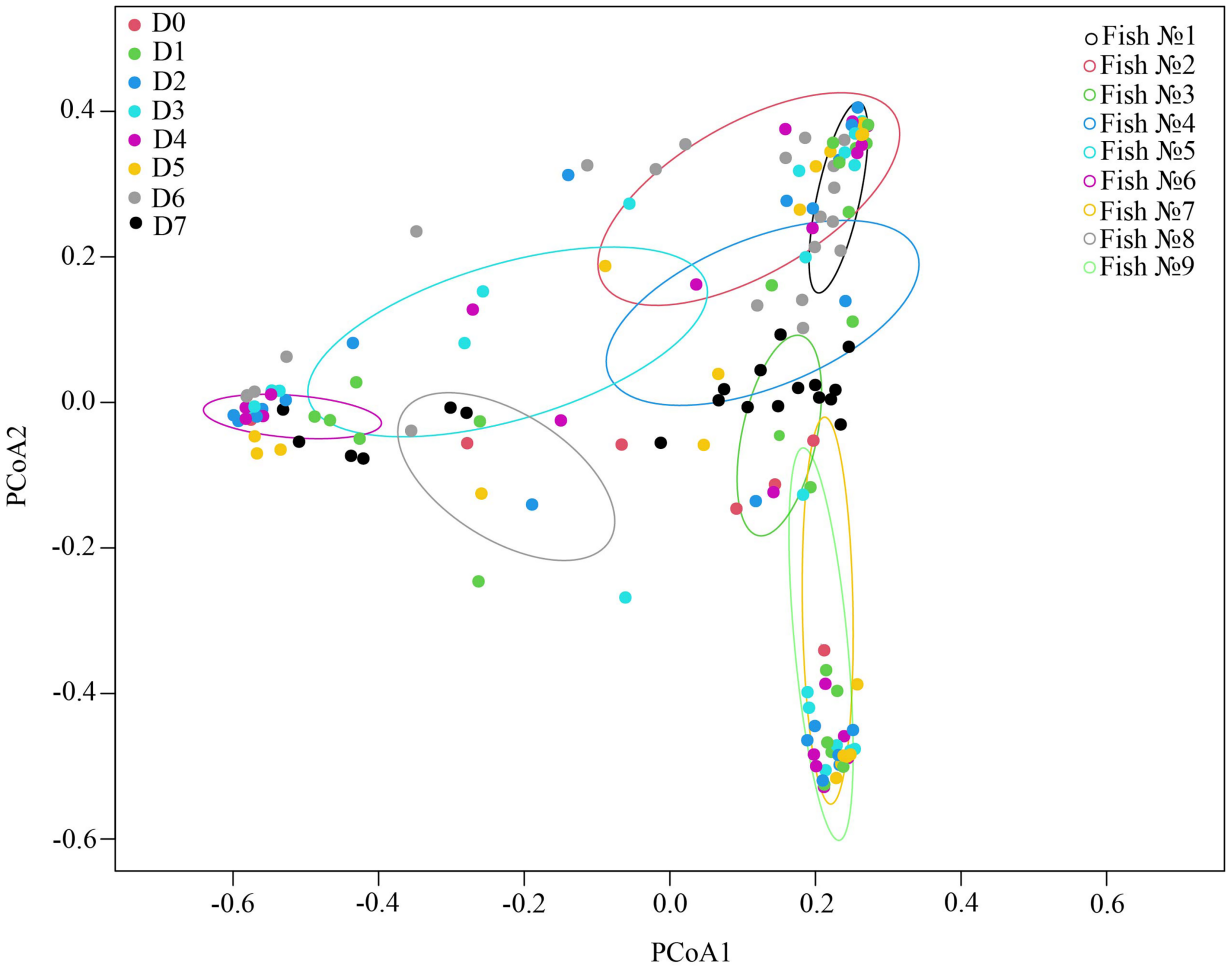
Test effect of factor «Fish» on microbial community of cestodes using ADONIS test on Weighted UniFrac matrix.

According to the ADONIS test based on Bray-Curtis matrix (Table 3) and using PCoA (Figure 5), significant differences in associated microbiota were found between all parts of the DT (stomach, anterior and posterior intestine) of infected and uninfected fish, and cestodes of different fractions (*p* < 0.05). Test effect of host-related factor «Fish» on the microbial community of cestodes using the ADONIS test and PCoA on Weighted UniFrac matrix (Supplementary Table S4) was also applied to degree of differences/similarity of microbiota of cestodes and the host in which they parasitize. The PCoA demonstrated a higher similarity of the microbial community of cestodes and their host (Figure 6). This means that for each host and their resident cestodes parasitizing their intestine there exists a unique microbial community that is significantly different, depending on the individual fish (ADONIS, *p* < 0.05).

**Table 3:** Comparison of the associated microbiota between different types of samples using ADONIS test based on Bray-Curtis matrix.

Combination			Uninfected “normal” *C. l. pidschian*		Infected “normal” *C. l. pidschian*	
			R2	*P*-value corrected	R2	*P*-value corrected
D0	vs.	Stomach content	0.15	**<0.0001**	0.19	**<0.0001**
		Stomach mucosa	0.15	**<0.0001**	0.21	**<0.0001**
		Washout from stomach mucosa	0.21	**<0.0001**	0.21	**0.001**
		Anterior content	0.09	**0.010**	0.13	**0.001**
		Anterior mucosa	0.14	**0.002**	0.14	**0.006**
		Washout from anterior mucosa	0.40	**<0.0001**	0.16	0.071
		Posterior content	0.10	**0.002**	0.14	**0.001**
		Posterior mucosa	0.19	**<0.0001**	0.21	**0.005**
		Washout from posterior mucosa	0.28	**0.001**	0.32	**0.001**
D1	vs.	Stomach content	0.17	**<0.0001**	0.19	**<0.0001**
		Stomach mucosa	0.16	**<0.0001**	0.19	**<0.0001**
		Washout from stomach mucosa	0.21	**<0.0001**	0.19	**<0.0001**
		Anterior content	0.15	**<0.0001**	0.16	**<0.0001**
		Anterior mucosa	0.14	**<0.0001**	0.13	**<0.0001**
		Washout from anterior mucosa	0.33	**<0.0001**	0.11	**0.013**
		Posterior content	0.15	**<0.0001**	0.18	**<0.0001**
		Posterior mucosa	0.18	**<0.0001**	0.14	**0.001**
		Washout from posterior mucosa	0.24	**<0.0001**	0.23	**<0.0001**
D2	vs.	Stomach content	0.15	**<0.0001**	0.17	**<0.0001**
		Stomach mucosa	0.15	**<0.0001**	0.18	**<0.0001**
		Washout from stomach mucosa	0.20	**<0.0001**	0.17	**<0.0001**
		Anterior content	0.15	**<0.0001**	0.14	**<0.0001**
		Anterior mucosa	0.15	**<0.0001**	0.12	**0.003**
		Washout from anterior mucosa	0.30	**<0.0001**	0.08	0.061
		Posterior content	0.14	**<0.0001**	0.18	**<0.0001**
		Posterior mucosa	0.16	**<0.0001**	0.13	**0.005**
		Washout from posterior mucosa	0.22	**<0.0001**	0.21	**<0.0001**
D3	vs.	Stomach content	0.17	**<0.0001**	0.18	**<0.0001**
		Stomach mucosa	0.15	**<0.0001**	0.18	**<0.0001**
		Washout from stomach mucosa	0.19	**<0.0001**	0.17	**<0.0001**
		Anterior content	0.15	**0.002**	0.15	**<0.0001**
		Anterior mucosa	0.15	**<0.0001**	0.12	**0.001**
		Washout from anterior mucosa	0.30	**<0.0001**	0.09	**0.038**
		Posterior content	0.15	**<0.0001**	0.19	**<0.0001**
		Posterior mucosa	0.17	**<0.0001**	0.13	**0.004**
		Washout from posterior mucosa	0.22	**<0.0001**	0.21	**<0.0001**
D4	vs.	Stomach content	0.15	**<0.0001**	0.16	**<0.0001**
		Stomach mucosa	0.14	**<0.0001**	0.17	**<0.0001**
		Washout from stomach mucosa	0.20	**<0.0001**	0.17	**<0.0001**
		Anterior content	0.14	**<0.0001**	0.14	**<0.0001**
		Anterior mucosa	0.14	**<0.0001**	0.12	**0.001**
		Washout from anterior mucosa	0.30	**<0.0001**	0.09	**0.031**
		Posterior content	0.14	**<0.0001**	0.17	**<0.0001**
		Posterior mucosa	0.16	**<0.0001**	0.12	**0.005**
		Washout from posterior mucosa	0.22	**<0.0001**	0.21	**<0.0001**
D5	vs.	Stomach content	0.15	**<0.0001**	0.16	**<0.0001**
		Stomach mucosa	0.13	**<0.0001**	0.17	**<0.0001**
		Washout from stomach mucosa	0.17	**<0.0001**	0.15	**<0.0001**
		Anterior content	0.14	**<0.0001**	0.14	**<0.0001**
		Anterior mucosa	0.13	**<0.0001**	0.12	**0.001**
		Washout from anterior mucosa	0.27	**<0.0001**	0.08	**0.046**
		Posterior content	0.14	**<0.0001**	0.17	**<0.0001**
		Posterior mucosa	0.15	**<0.0001**	0.11	**0.009**
		Washout from posterior mucosa	0.19	**<0.0001**	0.19	**<0.0001**
D6	vs.	Stomach content	0.22	**<0.0001**	0.24	**<0.0001**
		Stomach mucosa	0.22	**<0.0001**	0.27	**<0.0001**
		Washout from stomach mucosa	0.29	**<0.0001**	0.25	**<0.0001**
		Anterior content	0.23	**<0.0001**	0.22	**<0.0001**
		Anterior mucosa	0.25	**<0.0001**	0.21	**<0.0001**
		Washout from anterior mucosa	0.40	**<0.0001**	0.14	**0.008**
		Posterior content	0.22	**<0.0001**	0.27	**<0.0001**
		Posterior mucosa	0.24	**<0.0001**	0.20	**<0.0001**
		Washout from posterior mucosa	0.31	**<0.0001**	0.30	**<0.0001**
D7	vs.	Stomach content	0.24	**<0.0001**	0.26	**<0.0001**
		Stomach mucosa	0.22	**<0.0001**	0.25	**<0.0001**
		Washout from stomach mucosa	0.28	**<0.0001**	0.26	**<0.0001**
		Anterior content	0.18	**<0.0001**	0.23	**<0.0001**
		Anterior mucosa	0.15	**<0.0001**	0.17	**<0.0001**
		Washout from anterior mucosa	0.42	**<0.0001**	0.15	**0.004**
		Posterior content	0.20	**<0.0001**	0.24	**<0.0001**
		Posterior mucosa	0.24	**<0.0001**	0.20	**<0.0001**
		Washout from posterior mucosa	0.31	**<0.0001**	0.32	**<0.0001**

The bold character indicates significance at *p* < 0.05.

### Linear discriminant analysis effect size

**Figure 7:**
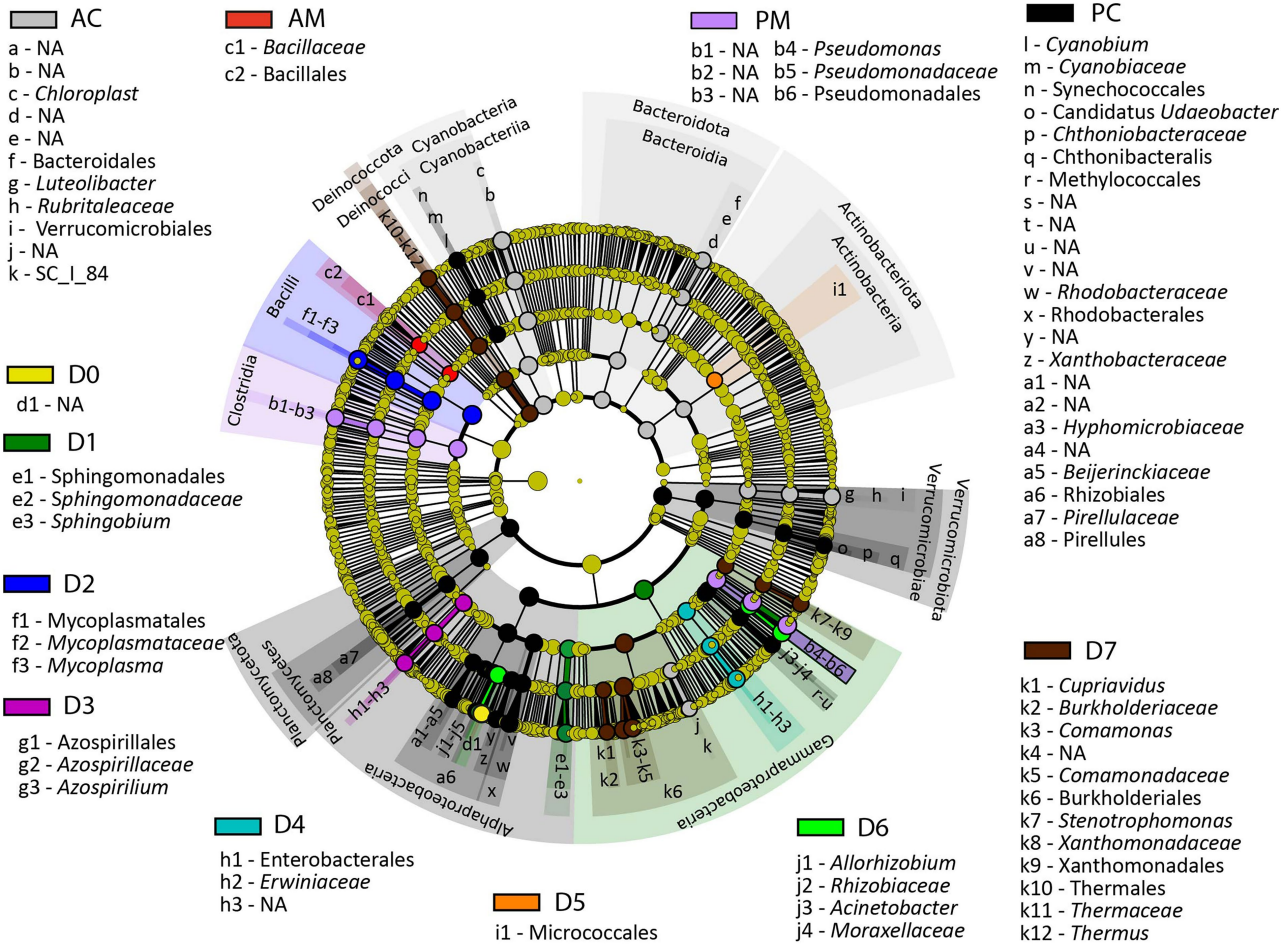
LEfSe results presenting the identified ASV that showed significant differences in abundances between the analyzed groups. AC, anterior content; PC, posterior content; AM, anterior mucosa; PM, posterior mucosa; NA, not identified.

The Linear discriminant analysis effect size (LEfSe) was performed to identify the bacterial ASV that showed significant differences in relative abundances among the analyzed groups (Figure 7 and Supplementary Table S5). The results show that *Sphingobium* was significantly different in the microbiota of the D1 fraction (LDA score = 4.95, *p* = 5.10E-03), whereas *Mycoplasma*, *Azospirillium*, *Erwiniaceae*, Micrococcales were significantly different in the microbiota of the D2 (LDA score = 5.31, *p* = 2.80E-03), D3 (LDA score = 4.52, *p* = 4.70E-07), D4 (LDA score = 4.67, *p* = 2.20E-11), and D5 fractions (LDA score = 4.04, *p* = 1.40E-04), correspondingly. In microbiota of the D6 fraction the significantly different bacterial taxa, compared to the other analyzed groups, were *Acinetobacter*, and *Allorhizobium* (LDA score = 5.10, *p* = 1.40E-07 and LDA score = 4.75, *p* = 4.10E-10, correspondingly). In microbiota of the D7 fraction the significantly different bacterial taxa were *Comamonas*, *Cupriavidus*, *Stenotrophomonas*, and *Thermus* (LDA score = 4.92, *p* = 2.49E-08, LDA score = 4.02, *p* = 8.00E-07, LDA score = 4.35, *p* = 1.30E-10, and LDA score = 4.31, *p* = 9.00E-08, correspondingly).

Compared to the other analyzed groups *Chloroplast*, *Luteolibacter*, and SC_I_84 from Betaproteobacteria were significantly different in content from the anterior intestine (LDA score = 4.94, *p* = 2.00E-17, LDA score = 4.55, *p* = 1.40E-22, and LDA score = 4.09, *p* = 1.20E-23, correspondingly), whereas unclassified Bacillales was significantly abundant in mucosa from the anterior intestine (LDA score = 4.01, *p* = 4.10E-05). In microbiota of posterior content, the significantly different bacterial taxa, compared to the other analyzed groups, were *Cyanobium* (LDA score = 4.16, *p* = 4.60E-19), Candidatus *Udaeobacter* (LDA score = 4.02, *p* = 5.10E-25), *Chthonibacteraceae* (LDA score = 4.28, *p* = 2.40E-23), Methylococcales (LDA score = 4.05, *p* = 3.50E-25), *Rhodobacteraceae* (LDA score = 4.59, *p* = 6.60E-21), *Xanthobacteraceae* (LDA score = 4.21, *p* = 6.80E-17), *Hyphomicrobiaceae* (LDA score = 4.10, *p* = 2.40E-22), *Beijerinckiaceae* (LDA score = 4.16, *p* = 1.20E-16), Rhizobiales (LDA score = 4.37, *p* = 1.90E-25), and Pirellules (LDA score = 4.28, *p* = 1.50E-23), whereas in posterior mucosa the significantly different bacterial taxa were *Pseudomonas* (LDA score = 5.38, *p* = 6.90E-07).

## Discussion

### Associated microbiota of different forms/species of whitefish

In the present study we have found significant differences in composition of the bacterial community between a small “dwarf” planktivorous and a large “normal” benthivorous forms/species. There is limited available data regarding the diversity of microbial communities in various sympatric pairs of salmonids (Sevellec et al., 2014, 2018, 2019; Belkova et al., 2017; Solovyev et al., 2019; Element et al., 2021). The microbiota of the intestinal mucosa of a sympatric pairs of *C. clupeaformis* was significantly different between “dwarf” and “normal” forms (Sevellec et al., 2018). For “dwarf” whitefish the genera *Stenotrophomonas*, and *Spartobacteria* were observed, whereas for the “normal” form of whitefish the bacteria from genera *Mycoplasma*, *Sarcina*, and *Serratia* were more abundant. At the same time the transient intestinal microbiota from the alimentary bolus obtained by Sevellec with co-authors (2019) in the same sympatric pairs of whitefish contained six dominant bacterial taxa: *Acinetobacter*, *Aeromonas*, *Clostridium*, *Legionella*, *Methylobacterium*, and *Propionibacterium*. According to these results the authors concluded that the adherent microbiota is more preferable to study the effect of host species on gut microbiota than the analysis of transient microbiota. The major drawback of this approach was discussed by Solovyev with co-authors (2019), where they concluded that rinsing of intestine with sterile saline solution could eliminate the bacteria with weak adherence to their mucosa and thus biasing further analysis. Due to the lack of conclusive data regarding the methodology for collecting samples of the digestive tract, we used a more comprehensive approach to analyze gastrointestinal microbiota of fish, where the stomach, anterior and posterior intestine was subdivided to the mucosal layer and their content with a parallel study of the washout from their mucosa. As a result, significant differences were obtained between mucosa from anterior and posterior intestine and their washout in uninfected “normal” and infected “dwarf” whitefish in comparison with infected fish. These results can be explained by the fact that the cestode, *Proteocephalus* sp. infected the anterior intestine and pyloric caeca of whitefish whereas in the posterior intestine these worms were almost absent. We assumed, that live worms are constantly moving in the fish intestine due to the different layers within the intestinal content and mucus continuously being added. In addition to this, it is known that worms can secret some molecules which induce the contraction of intestinal wall musculature (Saari et al., 2019). In parts of the intestine that are free from these parasites there is only intestinal contractions for mixing of layers, which is apparently not enough for deeply mixing different parts of the microbial community from intestinal mucus and content. Hence in uninfected parts, the difference in microbiota composition is significant between washout and mucosa due to lack of mixing by parasite movements. These results also indicate that the analysis of washing bacteria from the mucosa is more useful if it is necessary to assess the weakly associated intestinal microbiota.

A comparison of a sympatric pair of whitefish obtained in a previous study (Solovyev et al., 2019) to the present data, have shown that more stable microbial communities of whitefishes were observed in mucosa than in content. Thus, the shared bacteria at the lowest taxonomical level in the microbiota of “dwarf” whitefish mucosa were *Acinetobacter*, and *Mycoplasma*, whereas the shared bacteria in the microbiota of “normal” whitefish were *Acinetobacter, Aeromonas*, *Aeromonadaceae*. Microbiota of contents of both whitefishes analyzed by Solovyev with co-authors (2019) and in this study were different. These differences can be explained by fluctuations of the surrounding microbial community over lengths of time.

In a study similar to the current work, a comparison of a sympatric pair of whitefish from Canada analyzed microbiome samples from different sites among pooled samples obtained from *Salvelinus alpinus* and *C. clupeaformis* (Element et al., 2021). In that study, while it was noted that nearly half the samples had infection with cestodes (among other parasite), there was no separation of the parasites from the host intestinal microbiota analyzed. The microbial profiles included members of the genera *Mycoplasma* and *Deefgea* as seen with the cestode samples herein. Some of the microbes therefore described in the study by Element et al. (2021) may in fact be restricted to the cestodes and other intestinal parasites noted. This is not to say that these taxa have no effect on the host, but it does call into question what constitutes the “normal core microbiome” of the host (Margarita et al., 2016).

In the present study the parasite infection has significantly affected the microbial communities of the stomach and intestinal content of “normal” whitefish. Apparently, the gut tapeworms may have an effect on feeding regime and/or diet of infected fish, hence, such changes are reflected in the microbial composition. Indeed, it was shown that during co-infections (*F. psychrophilum*, *Renibacterium salmoninarum*, and ectoparasite *Caligus lacustris*) in rainbow trout, *Oncorhynchus mykiss* the gut of unhealthy fish was almost empty (Parshukov et al., 2019). The changes in gut microbiota of zebrafish *Danio rerio* during experimental *Pseudocapillaria tomentosa* infection was also revealed (Gaulke et al., 2019).

In this study, among “normal” whitefish there was a notable increased abundance of *Mycoplasma* in the cestode-infected fish, but in the uninfected “normal” whitefish there was a clear increase in *Aeromonas* and reduction in *Mycoplasma*. *Aeromonas* has been described previously as a dominant OTU of the “core microbiome” from multiple fishes with different feeding habits in nature (Ofek et al., 2021). The appearance of *Aeromonas* as a dominant OTU in wild-caught whitefish has been noted previously as well (Sevellec et al., 2019). The abundance of *Mycoplasma* in the cestode samples (D0-D7) and also in cestode-infected whitefish strongly suggests, in this context, that this represents a dysbiosis due to the infection by the cestodes. While cestodes may co-evolve with their hosts they may also be responsible for causing dysbiosis. However, curiously a study of *Mycoplasma* metagenomes collected from salmonids (among which whitefish are inclusive) suggested an underlying host benefit that is provided by *Mycoplasma* species; namely that their presence in the gut enables the juvenile salmonid to digest prey items enriched in long-chain polymers, such as chitin, which is often abundant in insects and crustaceans as a typical diet of whitefishes and the bacteria help to detoxify ammonia as well (Rasmussen et al., 2021). It has also been suggested that the presence of Mycoplasmas may be mutually exclusive for the presence of some potentially pathogenic *Vibrio* species (Rasmussen et al., 2021). These data could suggest that the abundance of Mycoplasmas is stage-specific for the fish host, and perhaps the abundance of *Aeromonas* is a more normal state for healthy adult whitefish. Additionally, studies with larger data sets are needed to improve clarity of this relationship.

### Associated microbiota of cestodes

Microbiota and parasitic helminths also interact with each other and sharing the same niches within the fish host (Cortés et al., 2019). Parasite-associated microbiota have been described in different classes of Platyhelminthes (Turbellaria, Monogenea, Trematoda, Cestoda). Among these associations some bacteria are present on the surface of the parasites (Cusack and Cone, 1985; Hughes-Stamm et al., 1999; Poddubnaya and Izvekova, 2005; Korneva and Plotnikov, 2006) and can be classified as ectosymbionts. Other bacteria that are present in helminth symbiotic organs (Gruber-Vodicka et al., 2011; Leisch et al., 2011; Caira and Jensen, 2021), and intestinal tract (Jorge et al., 2020, 2021) are called endosymbiotic bacteria.

Bacterial associations with tapeworms are especially interesting because this group of platyhelminths lacks all elements of a digestive system except absorption. During co-evolution, intestinal cestodes adapted to the microenvironment of their host and use it as a resource for low molecular weight nutrients. It is known that the external surfaces of tapeworms are composed of a multifunctional syncytial tegument performing digestive-absorptive functions which are similar in structure and function to the brush border of the intestines of vertebrates (Halton, 1997; Dalton et al., 2004). The first ultrastructural description of the presence of bacteria on the tegument surfaces of cestodes was made by Poddubnaya and Izvekova (2005). To date, the associated microbiota of cestodes has been studied using SEM and culture methods (Izvekova and Lapteva, 2004; Poddubnaya and Izvekova, 2005; Korneva and Plotnikov, 2006; Caira and Jensen, 2021).

Thus far there are only a few studies that have used a 16S rRNA sequencing approach to analyze the microbiota associated with fish cestodes (Hahn and Dheilly, 2018; Kashinskaya et al., 2020; Brealey et al., 2022; Hahn et al., 2022). Hahn and Dheilly (2018) characterized the microbiota of the tapeworm *Schistocephalus solidus* collected from the body cavity of threespine stickleback. In this study, parasites were shaken in sterile PBS to collect the surface microbiota of the *S. solidus* cestodes using culture-dependent methods. After rinsing in PBS solution the homogenate of cestodes was also sequenced. According to obtained results, the authors suggested that *S. solidus* cestodes contain their own endomicrobiome showing the absence of cultivable bacteria on the surface and presence of *Polynucleobacter* as a dominant taxon in the homogenate of *S. solidus* (Hahn and Dheilly, 2018). The possible assumption of the absence of bacteria on the tegument surfaces of *S. solidus* can be explained by the presence of special attachment structures in bacteria, which help them to adhere to the tegument of the parasite and making release of the bacterial cells more difficult. Using the SEM, the clear evidence of a strong association of bacteria with the tegument of fish cestodes has been obtained. Attachment of the individual bacterial cells to the tegument surface of cestodes is carried out *via* a special holdfast structure (stalk-like tufts and filaments) (Poddubnaya and Izvekova, 2005). As for the internal microbiome described by Hahn and Dheilly (2018), if the observed bacteria were not represented by adherent external bacteria, they may have in fact been representing an endomicrobiome.

Brealey with co-authors (2022) also characterized the microbial communities of *Eubothrium* cestode from the intestine of Atlantic salmon *Salmo salar*. In this study, parasites were also shaken in sterile PBS to collect loosely attached resident and transient microbes from salmon gut and associated microbiota of cestode tegument, as well as strongly associated bacteria with cestode tegument surface or body after washing (Brealey et al., 2022). According to this study, the microbiota of cestode body were dominated by *Mycoplasma*, while the cestode’s washout were dominated by *Mycoplasma*, *Carnobacterium*, and *Photobacterium*. However, without any confirmation of the complete removal of bacteria from the surface of the cestode tegument after short-term shaken (20 s), the question whether the microbiota of cestode body is the surface microbiota which are strongly associated with tegument, or the microbiota of the internal body cavity remains open.

**Figure 8:**
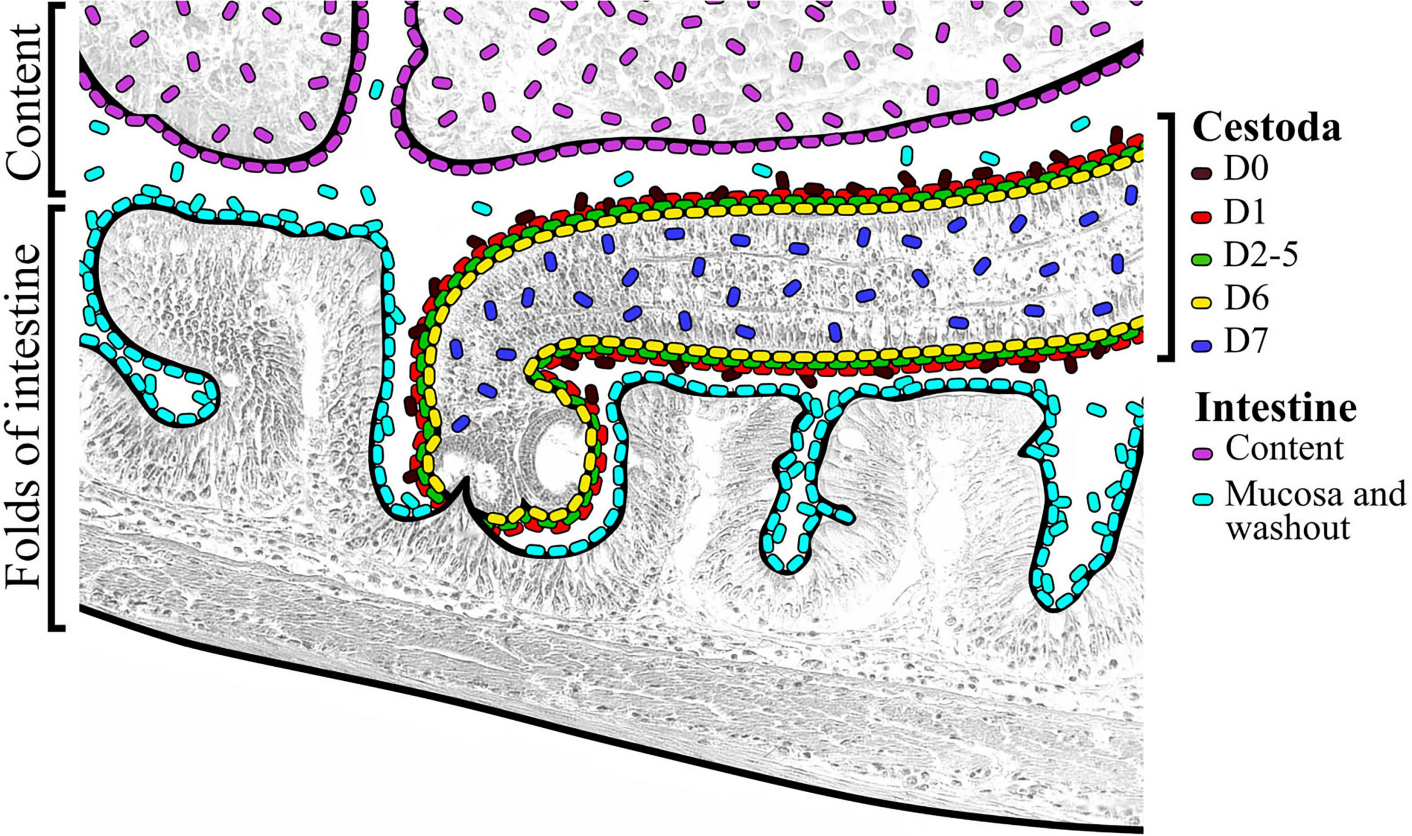
Schematic view of organization of different microbial communities associated with infected “normal” *C. l. pidschian* and the cestodes, *Proteocephalus* sp. parasitizing the intestine of fish.

In another study, Kashinskaya et al. (2020) estimated the structure of bacterial communities associated with the gastrointestinal tract of perch *Perca fluviatilis* with a parallel study of the microbiota associated with intestinal cestodes themselves. The bacteria from the genus *Mycoplasma*, *Serratia*, and *Pseudomonas* were the dominant taxa in the microbiota of cestodes of the genus *Proteocephalus* (Kashinskaya et al., 2020). Adding the “control” group (uninfected fish) and significantly improving the sample collection protocol for the cestode’s microbiota, we have analyzed the associated microbiota of the gut of whitefish as a complex multilevel system. According to the desorption method in Ringer’s solution, it was shown that for *Proteocephalus* sp. cestodes there are associations of several groups of microorganisms: (1) “Surface” microbiota of cestode’s (fraction D0), (2) weakly associated microbiota of cestodes (fraction D1), (3) microbiota strongly associated with the tegument (fraction D2-D5), (4) microbiota after treatment with detergent Triton X-100 (fraction D6), and (5) microbiota obtained after removal of the tegument from cestodes (D7) (Figure 8). The presence of bacteria after Triton X-100 treatment can be explained by the fact that cestodes have no digestive system, but they do have a reproductive tract and excretory organs (as noted above). Moreover, the specialized symbiotic organ in the form of infoldings of the dorsal and ventral surfaces of cestodes body *Elicilacunosus dharmadii* from eagle ray (*Aetomylaeus nichofii*) has been demonstrated to accommodate their bacterial symbionts (Caira and Jensen, 2021). Hughes-Stamm and co-authors identified 7 microbial morphotypes, including *Eubacteria*, and *Spirochaetes*, associated with the dorsal surface and excretory papillae regions of the trematode *Gyliauchenn nahaensis* isolated from *Siganus doliatus*, *S. orallines*, *S. puellus*, and *S. lineatus* (Hughes-Stamm et al., 1999). According to these findings we do not exclude the presence of bacteria in the reproductive tract of cestodes. The cestode gonopore connects the outside environment to internal cavities of the reproductive tract, such as the genital atrium below the surface of the tegument. As this is connected to the outside environment *via* the vagina and the common gonopore opening, though it is an internal space by definition, it is a region where the reproductive system is open to the external environment in the same way as our digestive system is open to the external environment. The presence of bacteria within the reproductive tract may not be a “normal” condition, but a type of infection of the tapeworm, just as vertebrates can get infections of their reproductive tract; however, it is also possible that this might be a “normal” microflora of the reproductive tract. Further validation of this finding is yet required.

Specific microenvironments of the intestine provide specific conditions for colonization by different groups of bacteria with a wide spectrum of functional and biochemical activity. The data from Figure 3 clearly shows a unique microbial signature from the cestodes as compared to the fish hosts. This is seen in the microbiome profiles from the separate fractions D0–D7. Among the distinct differences, several ASV are worth noting. *Acinetobacter*, *Azospirillum*, *Comamonas*, *Deefgea*, and *Sphingobium* are among those taxa more abundant in the cestode samples. The presence of these particular taxa may be indicating certain adaptations suitable for a symbiotic opportunist. *Acinetobacter* spp., are noted to possess a CRISPR/Cas system that positively influences biofilm production (Sarshar et al., 2021) that can in turn enhance persistence within the host, which may explain the close association with the cestode tegument in this study, predominating in fraction D6 (Supplementary Figure S2), even during removal and cleaning of the cestodes from fish gut. They are a group of species common in the natural environment, but are increasing in importance in human clinical settings due to increasing antibiotic resistance. From Supplementary Figure S2 we can see that much of *Sphingobium* is released early and also some *Comamonas*. This result is suggestive that these bacteria are not likely to be in the subsurface within the gonopore or parts of the reproductive tract. Others such *Mycoplasma* (which characterized by having very small cells and frequently exist as commensals due to their reduced genome) is more likely among cells that are located in more internal sites and removal and collection of these cells requires increasingly more stringent treatments. This persistence may be facilitated by biofilms or other mechanisms of adherence. The findings of *Mycoplasma* with parasites of the intestinal tract of fish is a possible indication of coevolution with this host with specific adaptations for survival such as receptor mediated surface attachment to the tegument or internal surfaces of the cestode (and/or the host). Symbiotic associations between cestodes and *Mycoplasma* have been noted previously (Margarita et al., 2016; Brealey et al., 2022) and so the benefits such as increased ATP production may also be at work imposing selective pressures on the host-pathogen relationship.

In summary, based on previous hypotheses we may make several conclusions: first, the rinsing procedure from the mucosa (as well as rinsing and shaking procedures for cestodes) are suitable approaches for broadly different ecological, biological, physiological, etc. studies, where detailed deep insight of the gut bacterial community structures and functions is needed because it permits separation of the microbial community into different subcommunities. But it has to be noted that this approach is based on features of adherence of different bacterial groups, and separation based on some other bacterial features (for example cell size, sedimentation velocity, or others) that may give different results. Since the aforementioned second, third, and fourth hypotheses were partially or completely supported because the gut microbiota of whitefish was affected by their cestode infection, thus, such factor as parasite infection has to be taken into account in studies focused on a “normal” vertebrate microbiome where different groups of matured parasites are also a normal part of the ecosystem. In addition, we hypothesize that, the presence of infected fish in a population will significantly increase the populations overall bacterial diversity that potentially may help them to resist environmental disturbances such as natural outbreaks of some diseases.

## Data availability statement

The datasets presented in this study can be found in online repositories. The names of the repository/repositories and accession number(s) can be found in the article/Supplementary material.

## Ethics statement

The animal study was reviewed and approved by the following information was supplied relating to ethical approvals (i.e., approving institutional body and any reference numbers): The present research has met the requirements guided by the order of the High and Middle Education Ministry (care for vertebrate animal included in scientific experiments, text number 742 from 13-11-1984) and additionally by the Federal Law of the Russian Federation text number 498 FL (from 19-12-2018) with regard to the humane treatment of animals.

## Author contributions

EK and MS conceived and designed the experiments, performed the experiments, analyzed the data, prepared figures and tables, contributed reagents, wrote the manuscript, and approved the final draft. ES performed the bioinformatics analysis, analyzed the data, prepared figures and tables, and approved the final draft. LP performed scanning and transmission electron microscopy, prepared figures, and approved the final draft. PV performed analysis of the 28S rRNA gene of *Proteocephalus* sp. and approved the final draft. AS designed graphs and prepared figures and tables. AP participated in the extraction of DNA from samples and approved the final draft. KA analyzed the data, wrote the manuscript, and approved the final draft. All authors contributed to the article and approved the submitted version.

## Funding

This work was supported by the Russian Science Foundation, project no. 19–74-00104 (analysis of the associated microbiota of cestodes before and after desorption), project no. 19–74-10054 (analysis of the associated microbiota of gastrointestinal tract of sympatric whitefishes), and the Russian international scientific collaboration program Mega-grant № 075-15-2022-1134 (analysis of the 28S rRNA gene of *Proteocephalus* sp. from whitefish).

## Conflict of interest

The authors declare that the research was conducted in the absence of any commercial or financial relationships that could be construed as a potential conflict of interest.

## Publisher’s note

All claims expressed in this article are solely those of the authors and do not necessarily represent those of their affiliated organizations, or those of the publisher, the editors and the reviewers. Any product that may be evaluated in this article, or claim that may be made by its manufacturer, is not guaranteed or endorsed by the publisher.

## Footnotes

^1^https://biolabmix.ru/upload/iblock/e59/BioMaster-HS_Taq-PCR_Color_full_eng.pdf

^2^https://support.illumina.com/documents/documentation/chemistry_documentation/16s/16s-metagenomic-library-prep-guide-15044223-b.pdf

^3^http://www2.decipher.codes/Classification/TrainingSets/SILVA_SSU_r138_2019.RData

^4^https://github.com/siberianhigh/miscellaneous/blob/main/pairwise.adonis2.FDR.R

^5^https://huttenhower.sph.harvard.edu/galaxy/

## Supplementary material

SUPPLEMENTARY FIGURE S1 Schematic view of sample collection. **(A)** Sympatric pair of whitefish inhibited the Lake Teletskoye (Russia): planktivorous *C. l. pravdinellus* and benthivorous *C. l. pidschian*. **(B)** Organizaton of gastriontestinal tract of different forms of whitefish. **(C)** Desorption of bacteria from tegument of cestodes.

SUPPLEMENTARY FIGURE S2 The relative abundances of main dominant of the microbial community associated with different fractions of cestodes. The lower-case character indicates significance at *p* ≤ 0.05 using Dunn’s test.
