# Corrigendum: Trophic diversification and parasitic invasion as ecological niche modulators for gut microbiota of whitefish

**DOI:** 10.3389/fmicb.2024.1363242

**Published:** 2024-02-13

**Authors:** Elena N. Kashinskaya, Evgeniy P. Simonov, Larisa G. Poddubnaya, Pavel G. Vlasenko, Anastasiya V. Shokurova, Aleksey N. Parshukov, Karl B. Andree, Mikhail M. Solovyev

**Affiliations:** ^1^Institute of Systematics and Ecology of Animals, Siberian Branch of the Russian Academy of Sciences, Novosibirsk, Russia; ^2^A.N. Severtsov Institute of Ecology and Evolution of the Russian Academy of Sciences, Moscow, Russia; ^3^Papanin Institute for Biology of Inland Waters, Russian Academy of Sciences, Yaroslavl Region, Russia; ^4^Institute of Biology of the Karelian Research Centre, Russian Academy of Sciences, Petrozavodsk, Russia; ^5^Institut de Recerca i Tecnologìa Agroalimentaries (IRTA), Sant Carles de la Ràpita, Spain; ^6^Tomsk State University, Biological Institute, Tomsk, Russia

**Keywords:** Coregonidae, microbiota, 16S rRNA sequencing, tegument of cestodes, desorption, scanning and transmission electron microscopy, electron microscopy

In the published article, there was an error in the Funding statement. The correct Funding statement appears below.

## Funding

This work was supported by the Russian Science Foundation, project no. 19–74-00104 (analysis of the associated microbiota of cestodes before and after desorption), project no. 19–74-10054 (analysis of the associated microbiota of gastrointestinal tract of sympatric whitefishes), and the Russian international scientific collaboration program Mega-grant Nō 075-15-2022-1134 (analysis of the 28S rRNA gene of *Proteocephalus* sp. from whitefish).

In the published article, Supplementary Figure S1 and Figure S2 was mistakenly not included in the publication. The missing material appears below:

Figure S1 and Figure S2.

In the published article, there was an error in the Materials and methods, *Study area and sampling*.

The corrected sentence appears below:

“Teletskoye Lake is a large (223 km^2^) and deep (325 m) oligotrophic lake (basin of Ob River) in the Altai Mountains (Altai Republic, Russia). In August 2019 in the north part of Teletskoye Lake (51.79°N; 87.30°E) “dwarf” C. l. pravdinellus (total length, TL 158.8 ± 2.6 mm, *n* = 14) infected by *Proteocephalus* sp., as well as “normal” C. l. pidschian uninfected (TL 252.2 ± 6.4 mm, *n* = 13) and infected by the same cestode (TL 241.3 ± 4.3 mm, *n* = 9) were collected (Supplementary Figure S1A and Supplementary Table S1). For microbiota investigations of “dwarf” whitefish we used only infected individuals due to the high prevalence level (100%) of *Proteocephalus* sp. Fish were captured using gill-nets (mesh sizes 18–25 mm) and transported alive to the laboratory in plastic containers filled with water from the site of fish capture. All fish were dissected and samples were collected aseptically. Male and female fish were identified according to gonadal development. The digestive tract (DT) was divided into three parts: stomach, anterior (without of pyloric caeca) and posterior intestine and cut separately (Supplementary Figure S1B). The content of each segment of DT were squeezed out by gentle stripping and collected separately. After collecting the content from the corresponding part of DT, the washing procedure was performed with sterile physiological saline solution (0.9% NaCl) to collect weakly adherent microbiota from mucosa of the stomach, anterior and posterior intestine of analyzed fish. Five milliliters of the solution were taken by syringe and slowly squeezed out into the “proximal” part of a vertically fixed part of the DT (stomach, anterior or posterior intestine), then when the solution passed through this part of the DT the solution was collected in an empty sterile tube at the “distal end” part of the DT. Afterward, the collected solution (washout) was stored at −80°C until analysis. After washing procedure, the upper mucosa from each segment of DT was then scraped off with a sterilized spatula and collected separately in another a sterile microcentrifuge tube.”

**Supplementary Figure S1:**
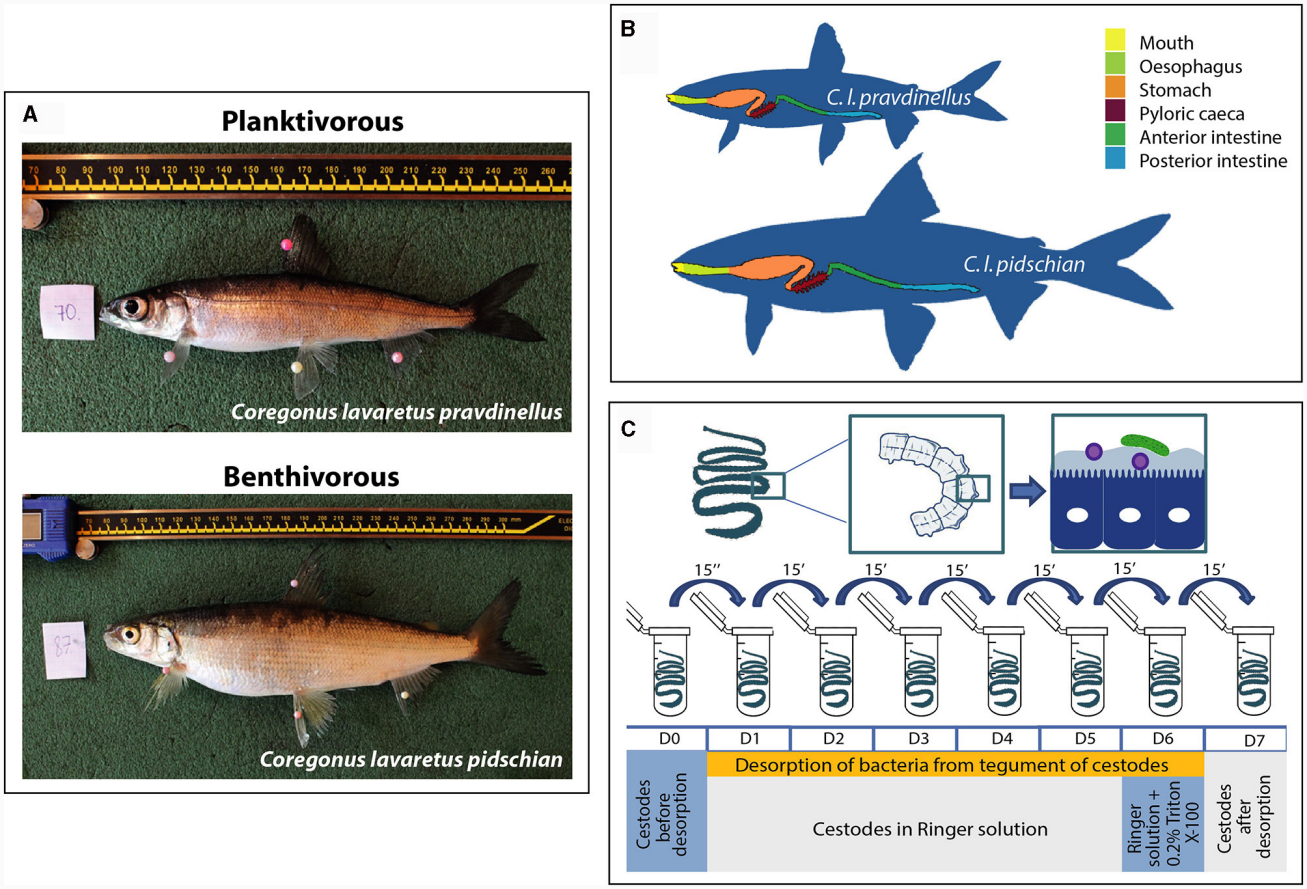
Schematic view of sample collection. **(A)** Sympatric pair of whitefish inhibited the Lake Teletskoye (Russia): planktivorous *C. l. pravdinellus* and benthivorous *C. l. pidschian*. **(B)** Organizaton of gastriontestinal tract of different forms of whitefish. **(C)** Desorption of bacteria from tegument of cestodes.

**Supplementary Figure S2:**
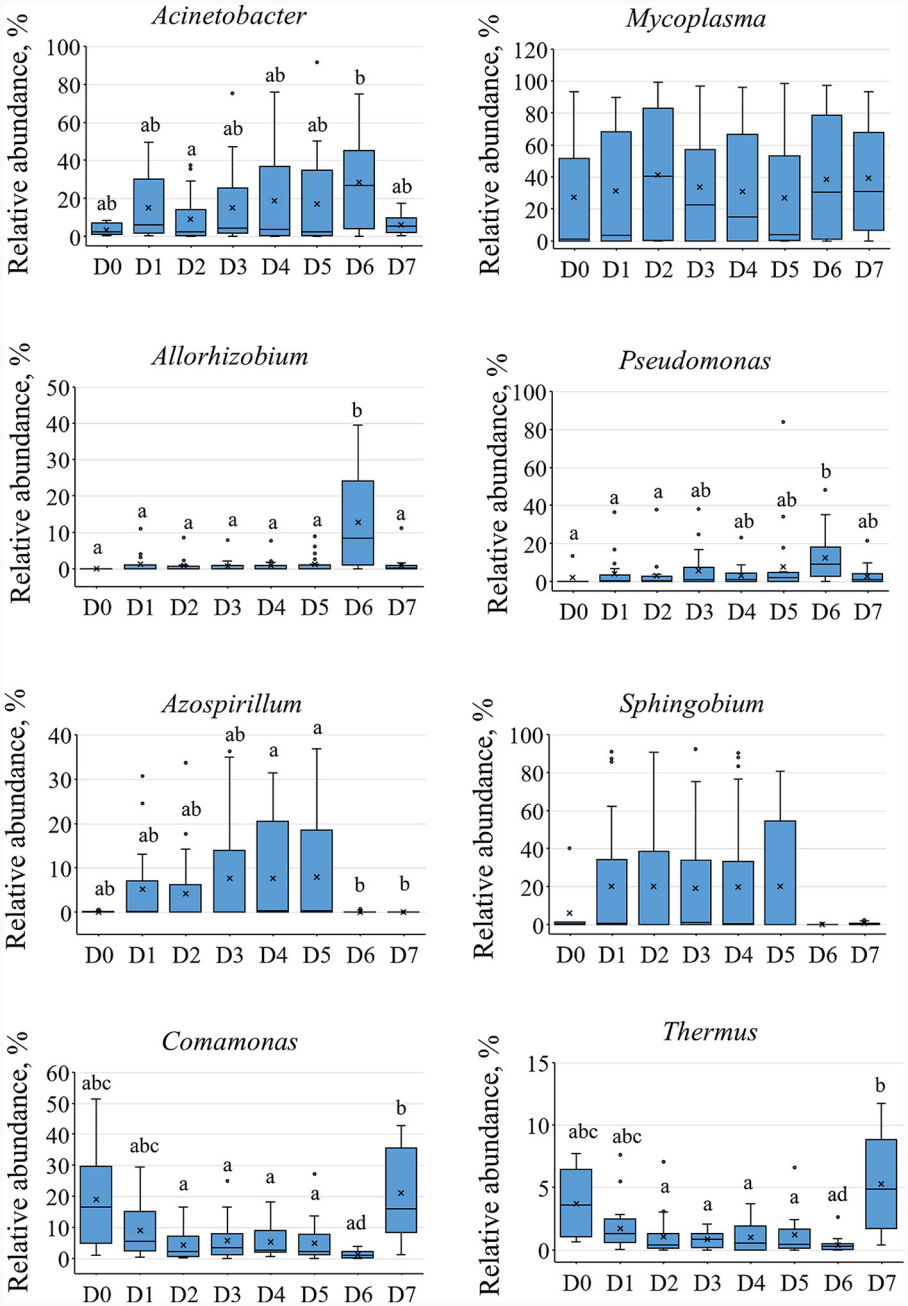
The relative abundances of main dominant of the microbial community associated with different fractions of cestodes. The lower-case character indicates significance at *p* ≤ 0.05 using Dunn's test.

The authors apologize for these errors and state that this does not change the scientific conclusions of the article in any way. The original article has been updated.

