# Supplementary figures and images for: Trophic diversification and parasitic invasion as ecological niche modulators for gut microbiota of whitefish
Source: Front Microbiol. 2023 Mar 14;14:1090899. doi: 10.3389/fmicb.2023.1090899 (PMC10043260; doi:10.3389/fmicb.2023.1090899)

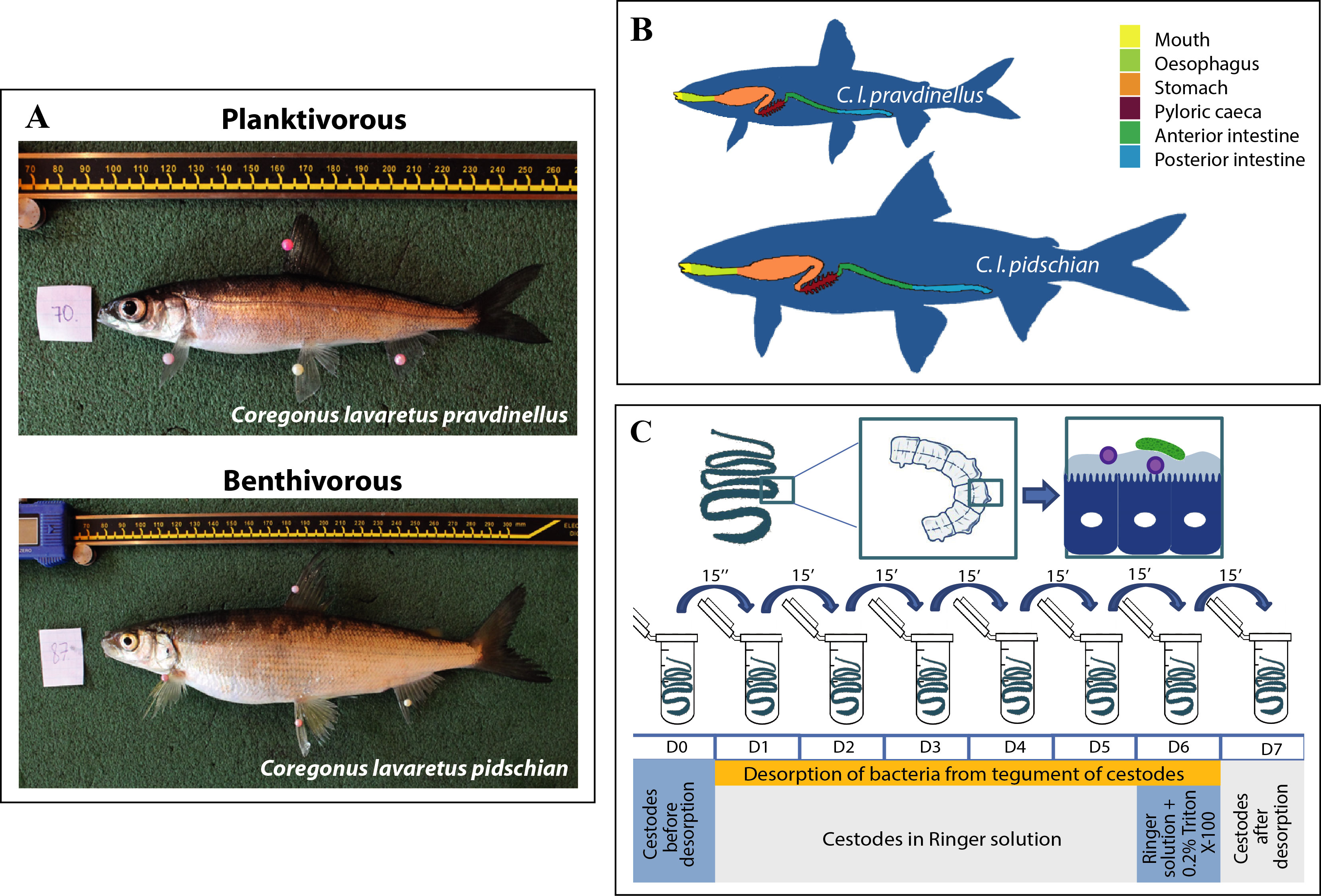

Supplement: SUPPLEMENTARY FIGURE S1 — Schematic view of sample collection. (A) Sympatric pair of whitefish inhibited the Lake Teletskoye (Russia): planktivorous C. l. pravdinellus and benthivorous C. l. pidschian. (B) Organizaton of gastriontestinal tract of different forms of whitefish. (C) Desorption of bacteria from tegument of cestodes. [file Figure_1.JPEG]

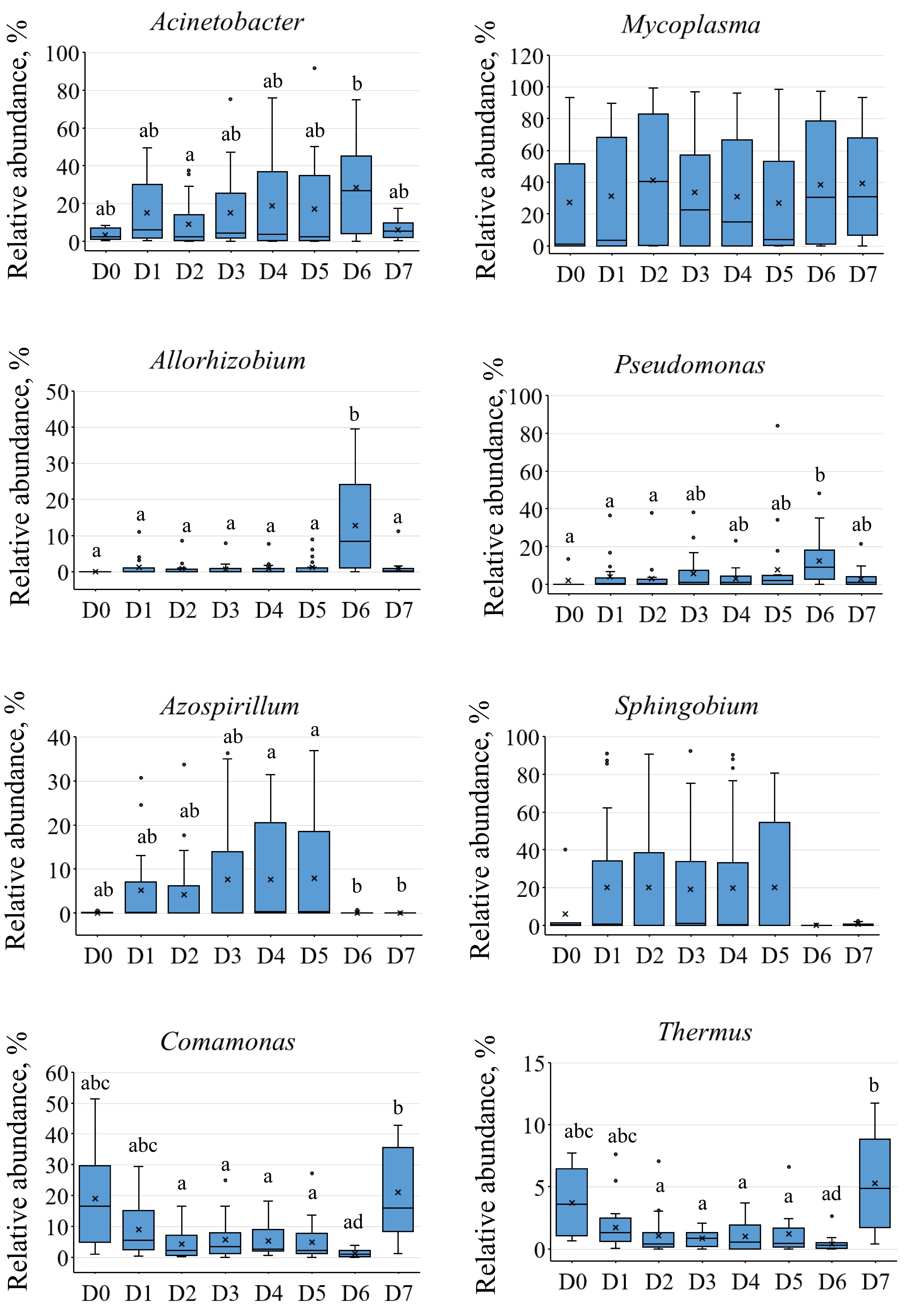

Supplement: SUPPLEMENTARY FIGURE S2 — The relative abundances of main dominant of the microbial community associated with different fractions of cestodes. The lower-case character indicates significance at p ≤ 0.05 using Dunn’s test. [file Figure_2.JPEG]
