# Supplementary material for: Trophic diversification and parasitic invasion as ecological niche modulators for gut microbiota of whitefish
Source: Front Microbiol. 2023 Mar 14;14:1090899. doi: 10.3389/fmicb.2023.1090899 (PMC10043260; doi:10.3389/fmicb.2023.1090899)
Supplement: Supplementary file 3 [file Data_Sheet_1.PDF]

Bioinformatic processing of raw 16S rRNA read data

Cutadapt v. 4.1 (Martin, 2011) was used on raw demultiplexed reads to trim primer sequences from both forward and reverse reads with the primer finding and removal step repeated two times and discarding reads do not containing primer sequences:

```
for sample in $(cat samples)
do
    echo "On sample: $sample"
        cutadapt -a CCTACGGGNGGCWGCAG...GGATTAGATACCCBDGTAGTC \
        -A GACTACHVGGGTATCTAATCC...CTGCWGCCNCCCGTAGG \
        -m 170 -e 0.2 --times 2 --discard-untrimmed -j 4 \
        -o ${sample}_R1_trimmed.fq.gz -p ${sample}_R2_trimmed.fq.gz \
        ${sample}_R1.fq ${sample}_R2.fq \
        >> cutadapt_primer_trimming_stats.txt 2>&1
  done
```

DADA2 v. 1.24 pipeline (Callahan et al., 2016) was used to process raw 16S reads into amplicon sequence variants (ASVs) at the 100% nucleotide identity. Create sample variables:

```
library(dada2)

samples <- scan("samples", what="character")
forward_reads <- paste0(samples, "_R1_trimmed.fq.gz")
reverse_reads <- paste0(samples, "_R2_trimmed.fq.gz")
filtered_forward_reads <- paste0(samples, "_R1_filtered.fq.gz")
filtered_reverse_reads <- paste0(samples, "_R2_filtered.fq.gz")
```

Plotting quality profiles to decide on trimming thresholds:

```
plotQualityProfile(forward_reads, aggregate = TRUE)
plotQualityProfile(reverse_reads, aggregate = TRUE)
```

Forward reads were trimmed by 15 bp on the 5’ end and truncated at position 250, while reverse reads were truncated at position 200. We also discarded any reads contained more than two expected errors:

```
filtered_out <- filterAndTrim(forward_reads, filtered_forward_reads,
                    reverse_reads, filtered_reverse_reads, maxEE=c(2,2),
                    rm.phix=TRUE, minLen=150, trimLeft=c(15,0), truncLen=c(250,200),
                    multithread = TRUE, verbose = TRUE)
```

The minimum number of total bases to use for error rate learning was set to 10^9 with randomization allowed:

```
err_forward_reads <- learnErrors(filtered_forward_reads, nbases = 1e+09, randomize=TRUE,
multithread=TRUE)
err_reverse_reads <- learnErrors(filtered_reverse_reads, nbases = 1e+09, randomize=TRUE,
multithread=TRUE)

plotErrors(err_forward_reads, nominalQ=TRUE)
plotErrors(err_reverse_reads, nominalQ=TRUE)
```

Then the reads were dereplicated, denoised and merged with default parameters:

```
dada_forward_ind <- dada(filtered_forward_reads, err=err_forward_reads, multithread=TRUE)
dada_reverse_ind <- dada(filtered_reverse_reads, err=err_forward_reads, multithread=TRUE)
merged_amplicons <- mergePairs(dada_forward_ind, filtered_forward_reads, dada_reverse_ind,
filtered_reverse_reads, trimOverhang=TRUE, verbose=TRUE)
```

Resulting ASVs shorter that 350 bp were discarded:

```
seqtab <- makeSequenceTable(merged_amplicons)
seqtable <- seqtab[,nchar(colnames(seqtab)) %in% 350:450]
```

Chimeric sequences removal:

```
seqtab.nochim <- removeBimeraDenovo(seqtab, method="consensus", multithread=TRUE,
verbose=TRUE)
```

The IDTAXA algorithm (Murali et al., 2018) of the DECIPHER v. 2.24 R package (Wright, 2016) was used to assign taxonomy to each ASV with training set SILVA SSU r138 (modified) (http://www2.decipher.codes/Classification/TrainingSets/SILVA_SSU_r138_2019.Rdata):

```
library(DECIPHER)
load("./SILVA_SSU_r138_2019.RData")
dna <- DNAStringSet(getSequences(seqtab.nochim)) # creating DNAStringSet object of ASVs
# classifying
tax_info <- IdTaxa(test=dna, trainingSet=trainingSet, strand="both", processors=NULL)
```

Create output tables with ASVs counts and taxonomy:

```
# giving more manageable names to sequence headers
asv_seqs <- colnames(seqtab.nochim)
asv_headers <- vector(dim(seqtab.nochim)[2], mode="character")

for (i in 1:dim(seqtab.nochim)[2]) {
  asv_headers[i] <- paste(">ASV", i, sep="_")
}

# count table:
asv_tab <- t(seqtab.nochim)
row.names(asv_tab) <- sub(">", "", asv_headers)
write.table(asv_tab, "ASVs_counts.tsv", sep="\t", quote=F, col.names=NA)

# creating table of taxonomy and setting any that are unclassified as "NA"
ranks <- c("domain", "phylum", "class", "order", "family", "genus", "species")
asv_tax <- t(sapply(tax_info, function(x) {
  m <- match(ranks, x$rank)
  taxa <- x$taxon[m]
  taxa[startsWith(taxa, "unclassified_")] <- NA
  taxa
}))
colnames(asv_tax) <- ranks
```

```
rownames(asv_tax) <- gsub(pattern=">", replacement="", x=asv_headers)

ranks <- c("domain", "phylum", "class", "order", "family", "genus", "species")

# Convert the output object of class "Taxa" to a matrix analogous to the output from
assignTaxonomy

taxid <- t(sapply(tax_info, function(x) {
  m <- match(ranks, x$rank)
  taxa <- x$taxon[m]
  taxa[startsWith(taxa, "unclassified_")] <- NA
  taxa
}))
colnames(taxid) <- ranks; rownames(taxid) <- getSequences(seqtab.nochim)

# Make QIIME-like OTU table
asv_table <- cbind(as.data.frame(asv_tab), as.data.frame(asv_tax))
```

We retained for further analysis only ASVs assigned to Bacteria at least at Phylum level. All singleton and doubleton ASVs were filtered from the samples:

```
asv_table_b <- asv_table[ which(asv_table$domain == "Bacteria"), ] # keep Bacteria only
asv_table_bp <- asv_table_b[ which(new$phylum != "NA"), ] # remove Bacterial ASVs do not
assigned at Phylum level

good_asvs <- row.names(asv_table_bp)

# making new count table
asv_tab_good <- asv_tab[row.names(asv_tab) %in% good_asvs, ]

# making new taxonomy table
asv_tax_good <- asv_tax[row.names(asv_tax) %in% good_asvs, ]

# making new ASV table
asv_table_good <- asv_table[row.names(asv_tax) %in% good_asvs, ]

# add column with a number of reads per sample
asv_tab_good_rowSum <- as.data.frame(cbind(asv_tab_good , ASVSize =
rowSums(asv_tab_good )))

z <- 3  # desired threshold for a number of reads per ASV
df <- asv_tab_good_rowSum[asv_tab_good_rowSum$ASVSize >= z, ]

b <- ncol(asv_tab_good_rowSum)
asv_tab_final<- df[, -b]

good_asvs <- row.names(asv_tab_final)
write.table(asv_table_final, "ASVs_tab_final.tsv", sep = "\t", quote=F, col.names=NA)

# making final taxonomy table
asv_tax_final <- asv_tax_good[row.names(asv_tax_good) %in% good_asvs, ]
write.table(asv_tax_final, "ASVs_tax_final.tsv", sep = "\t", quote=F, col.names=NA)
```

```
# making final QIIME-like OTU table
asv_table_final <- cbind(as.data.frame(asv_tab_final), as.data.frame(asv_tax_final))
write.table(asv_table_final, "ASVs_table_final.tsv", sep = "\t", quote=F, col.names=NA)
```
