# Supplementary material for: Trophic diversification and parasitic invasion as ecological niche modulators for gut microbiota of whitefish
Source: Front Microbiol. 2023 Mar 14;14:1090899. doi: 10.3389/fmicb.2023.1090899 (PMC10043260; doi:10.3389/fmicb.2023.1090899)
Supplement: Supplementary file 5 [file Table_1.docx]

Table S1. Sample information.

| Form/species | Number of fish | Body weight, g | Body weight without viscera, g | Total length, mm | Standard length, mm | Sex |
| --- | --- | --- | --- | --- | --- | --- |
| ***Infected “dwarf” C. l. pravdinellus*** | 1 | 32.5 | 25.7 | 165.3 | 139.5 | M |
|  | 2 | 40.7 | 33.8 | 183.8 | 150.9 | M |
|  | 3 | 36.3 | 29.5 | 165.0 | 135.0 | M |
|  | 4 | 42.7 | 35.0 | - | - | M |
|  | 5 | 31.0 | 24.7 | 155.0 | 130.0 | M |
|  | 6 | 28.0 | 22.9 | 155.0 | 125.0 | M |
|  | 7 | 22.0 | 17.4 | 150.0 | 125.0 | M |
|  | 8 | 26.2 | 20.3 | 150.0 | 125.0 | M |
|  | 9 | 24.5 | 19.7 | 153.5 | 134.5 | M |
|  | 10 | 27.4 | 21.6 | 166.8 | 139.2 | M |
|  | 11 | 30.3 | 24.7 | 160.0 | 130.0 | M |
|  | 12 | 31.5 | 25.1 | 150.0 | 130.0 | M |
|  | 13 | 28.6 | 23.3 | 150.0 | 120.0 | M |
|  | 14 | 36.0 | 28.7 | 160.0 | 135.0 | F |
|  | **Mean±SE** | **31.3±1.6** | **25.2±1.4** | **158.8±2.6** | **132.2±2.2** |  |
| ***Uninfected “normal” C. l. pidschian*** | 1 | 136.7 | 116.3 | 264.4 | 227.3 | F |
|  | 2 | 218.8 | 187.5 | 295.9 | 245.7 | M |
|  | 3 | 182.2 | 159.8 | 290.3 | 240.9 | F |
|  | 4 | 176.0 | 152.7 | 260.6 | 221.7 | F |
|  | 5 | 108.3 | 92.6 | 231.2 | 195.1 | F |
|  | 6 | 127.8 | 111.9 | 244.3 | 205.8 | F |
|  | 7 | 120.0 | 103.5 | 230.0 | 190.0 | F |
|  | 8 | 134.7 | 116.2 | 253.4 | 124.0 | F |
|  | 9 | 124.1 | 108.2 | 225.0 | 190.0 | F |
|  | 10 | 144.0 | 125.6 | 235.0 | 200.0 | M |
|  | 11 | 108.0 | 93.1 | 230.0 | 190.0 | F |
|  | 12 | 226.5 | 196.6 | - | - | F |
|  | 13 | 137.4 | 120.7 | 265.8 | 222.2 | M |
|  | **Mean±SE** | **149.6±10.9** | **129.6±9.1** | **252.2±6.4** | **204.4±8.6** |  |
| ***Infected “normal” C. l. pidschian*** | 1 | 116.0 | 99.0 | 220.0 | 185.0 | F |
|  | 2 | 119.2 | 95.6 | 233.1 | 195.2 | F |
|  | 3 | 171.0 | 149.0 | 238.0 | 227.0 | M |
|  | 4 | 160.0 | 144.3 | 260.0 | 210.0 | M |
|  | 5 | 114.9 | 99.3 | 240.1 | 220.3 | M |
|  | 6 | 127.1 | 108.1 | 249.1 | 210.3 | F |
|  | 7 | 138.3 | 115.4 | 256.0 | 211.0 | M |
|  | 8 | 99.2 | 86.9 | 230.0 | 190.0 | M |
|  | 9 | 111.0 | 95.6 | 245.0 | 203.0 | F |
|  | **Mean±SE** | **128.5±7.9** | **110.4±7.4** | **241.3±4.3** | **205.8±4.6** |  |

Table S3. Comparison of alpha-and beta-diversity of microbial community between form/species of whitefish and different segment of their gastrointestinal tract. The bold character indicates significance at p<0.05.

| Combination | | | | Alpha-diversity | | | | | | | | | | | | Beta-diversity | | |
| --- | --- | --- | --- | --- | --- | --- | --- | --- | --- | --- | --- | --- | --- | --- | --- | --- | --- | --- |
|  |  |  |  | ASV | | | | Shannon | | | | Simpson | | | | ADONIS on Bray-Curtis | | |
|  |  |  |  | Z statistic | | Adjusted p-value | | Z statistic | | Adjusted p-value | | Z statistic | | Adjusted p-value | | R2 | | P-value corrected |
| Between different form/species of whitefish | | | | | | | | | | | | | | | | | | |
| Stomach content (*C. l. pravdinellus*) | vs. | Stomach content (uninfected *C. l. pidschian*) | -1.57 | | 0.099 | | -0.300 | | -0.424 | | 0.109 | | -0.474 | | 0.22 | | **0.000** | |
| Stomach mucosa (*C. l. pravdinellus*) | vs. | Stomach mucosa (uninfected *C. l. pidschian*) | -0.37 | | 0.393 | | -0.949 | | -0.254 | | -0.810 | | -0.305 | | 0.10 | | **0.004** | |
| Washout from stomach mucosa (*C. l. pravdinellus*) | vs. | Washout from stomach mucosa (uninfected *C. l. pidschian*) | 0.33 | | 0.403 | | 0.744 | | -0.309 | | 0.946 | | -0.270 | | 0.22 | | **0.000** | |
| Anterior content (*C. l. pravdinellus*) | vs. | Anterior content (uninfected *C. l. pidschian*) | -3.86 | | **0.000** | | -3.257 | | **0.005** | | -2.866 | | **0.017** | | 0.16 | | **0.000** | |
| Anterior mucosa (*C. l. pravdinellus*) | vs. | Anterior mucosa (uninfected *C. l. pidschian*) | -2.15 | | **0.036** | | -2.684 | | **0.021** | | -2.327 | | -0.044 | | 0.34 | | **0.000** | |
| Washout from anterior mucosa (*C. l. pravdinellus*) | vs. | Washout from anterior mucosa (uninfected *C. l. pidschian*) | 0.03 | | 0.489 | | 2.649 | | **0.022** | | 3.381 | | **0.006** | | 0.40 | | **0.000** | |
| Posterior content (*C. l. pravdinellus*) | vs. | Posterior content (uninfected *C. l. pidschian*) | -0.55 | | 0.342 | | 1.408 | | -0.157 | | 0.677 | | -0.339 | | 0.21 | | **0.000** | |
| Posterior mucosa (*C. l. pravdinellus*) | vs. | Posterior mucosa (uninfected *C. l. pidschian*) | 1.02 | | 0.213 | | 1.694 | | -0.109 | | 1.530 | | -0.147 | | 0.21 | | **0.000** | |
| Washout from posterior mucosa (*C. l. pravdinellus*) | vs. | Washout from posterior mucosa (uninfected *C. l. pidschian*) | 2.38 | | **0.021** | | 3.233 | | **0.005** | | 3.406 | | **0.005** | | 0.26 | | **0.000** | |
| Stomach content (*C. l. pravdinellus*) | vs. | Stomach content (infected *C. l. pidschian*) | -1.47 | | 0.116 | | -0.530 | | -0.365 | | -0.152 | | -0.466 | | 0.29 | | **0.001** | |
| Stomach mucosa (*C. l. pravdinellus*) | vs. | Stomach mucosa (infected *C. l. pidschian*) | -1.20 | | 0.172 | | -2.459 | | -0.030 | | -2.438 | | -0.038 | | 0.21 | | **0.000** | |
| Washout from stomach mucosa (*C. l. pravdinellus*) | vs. | Washout from stomach mucosa (infected *C. l. pidschian*) | -0.36 | | 0.393 | | 0.024 | | -0.494 | | 0.157 | | -0.466 | | 0.20 | | **0.000** | |
| Anterior content (*C. l. pravdinellus*) | vs. | Anterior content (infected *C. l. pidschian*) | -3.97 | | **0.000** | | -2.866 | | **0.013** | | -2.494 | | -0.035 | | 0.16 | | **0.000** | |
| Anterior mucosa (*C. l. pravdinellus*) | vs. | Anterior mucosa (infected *C. l. pidschian*) | -0.99 | | 0.221 | | -2.088 | | -0.056 | | -1.893 | | -0.089 | | 0.29 | | **0.000** | |
| Washout from anterior mucosa (*C. l. pravdinellus*) | vs. | Washout from anterior mucosa (infected *C. l. pidschian*) | 0.97 | | 0.224 | | 1.414 | | -0.156 | | 1.303 | | -0.190 | | 0.17 | | **0.005** | |
| Posterior content (*C. l. pravdinellus*) | vs. | Posterior content (infected *C. l. pidschian*) | -1.52 | | 0.107 | | -3.193 | | **0.005** | | -3.248 | | **0.008** | | 0.21 | | **0.000** | |
| Posterior mucosa (*C. l. pravdinellus*) | vs. | Anterior mucosa (infected *C. l. pidschian*) | 0.51 | | 0.353 | | 0.883 | | -0.269 | | 1.254 | | -0.201 | | 0.22 | | **0.001** | |
| Washout from posterior mucosa (*C. l. pravdinellus*) | vs. | Washout from posterior mucosa (infected *C. l. pidschian*) | -0.36 | | 0.393 | | 2.533 | | -0.027 | | 3.129 | | **0.010** | | 0.29 | | **0.000** | |
| Between uninfected and infected *C. l. pidschian* | | | | | | | | | | | | | | | | | | |
| Stomach content (infected *C. l. pidschian*) | vs. | Stomach content (uninfected *C. l. pidschian*) | 0.04 | | 0.488 | | 0.281 | | -0.427 | | 0.275 | | -0.437 | | 0.06 | | 0.253 | |
| Stomach mucosa (infected *C. l. pidschian*) | vs. | Stomach mucosa (uninfected *C. l. pidschian*) | 0.88 | | 0.252 | | 1.610 | | -0.121 | | 1.714 | | -0.113 | | 0.09 | | **0.016** | |
| Washout from stomach mucosa (infected *C. l. pidschian*) | vs. | Washout from stomach mucosa (uninfected *C. l. pidschian*) | 0.62 | | 0.323 | | 0.596 | | -0.346 | | 0.638 | | -0.344 | | 0.04 | | 0.864 | |
| Anterior content (infected *C. l. pidschian*) | vs. | Anterior content (uninfected *C. l. pidschian*) | 0.46 | | 0.369 | | -0.122 | | -0.473 | | -0.138 | | -0.469 | | 0.07 | | **0.029** | |
| Anterior mucosa (infected *C. l. pidschian*) | vs. | Anterior mucosa (uninfected *C. l. pidschian*) | -1.10 | | 0.192 | | -0.524 | | -0.365 | | -0.372 | | -0.409 | | 0.09 | | 0.089 | |
| Washout from anterior mucosa (infected *C. l. pidschian*) | vs. | Washout from anterior mucosa (uninfected *C. l. pidschian*) | -0.96 | | 0.226 | | 0.621 | | -0.340 | | 1.302 | | -0.189 | | 0.29 | | **0.012** | |
| Posterior content (infected *C. l. pidschian*) | vs. | Posterior content (uninfected *C. l. pidschian*) | 1.08 | | 0.197 | | 2.224 | | -0.046 | | 2.150 | | -0.060 | | 0.09 | | **0.005** | |
| Posterior mucosa (infected *C. l. pidschian*) | vs. | Posterior mucosa (uninfected *C. l. pidschian*) | 0.24 | | 0.430 | | 0.380 | | -0.410 | | -0.108 | | -0.474 | | 0.10 | | 0.231 | |
| Washout from posterior mucosa (infected *C. l. pidschian*) | vs. | Washout from posterior mucosa (uninfected *C. l. pidschian*) | 1.16 | | 0.177 | | 0.288 | | -0.425 | | -0.128 | | -0.472 | | 0.05 | | 0.783 | |
| Between mucosa and content | | | | | | | | | | | | | | | | | | |
| Stomach content (*C. l. pravdinellus*) | vs. | Stomach mucosa (*C. l. pravdinellus*) | 1.68 | | 0.085 | | 1.083 | | -0.219 | | 0.443 | | -0.396 | | 0.09 | | 0.085 | |
| Anterior content (*C. l. pravdinellus*) |  | Anterior mucosa (*C. l. pravdinellus*) | 0.98 | | 0.222 | | 1.505 | | -0.138 | | 1.558 | | -0.143 | | 0.14 | | **0.011** | |
| Posterior content (*C. l. pravdinellus*) |  | Posterior mucosa (*C. l. pravdinellus*) | 2.66 | | **0.011** | | -0.329 | | -0.418 | | -0.907 | | -0.282 | | 0.19 | | **0.000** | |
| Stomach content (uninfected *C. l. pidschian*) | vs. | Stomach mucosa (uninfected *C. l. pidschian*) | 3.27 | | **0.000** | | 0.598 | | -0.347 | | -0.436 | | -0.394 | | 0.09 | | **0.001** | |
| Anterior content (uninfected *C. l. pidschian*) | vs. | Anterior mucosa (uninfected *C. l. pidschian*) | 2.62 | | **0.012** | | 2.003 | | -0.065 | | 2.049 | | -0.068 | | 0.10 | | **0.004** | |
| Posterior content (uninfected *C. l. pidschian*) | vs. | Posterior mucosa (uninfected *C. l. pidschian*) | 4.28 | | **0.000** | | 2.596 | | **0.024** | | 1.979 | | -0.077 | | 0.10 | | **0.003** | |
| Stomach content (infected *C. l. pidschian*) | vs. | Stomach mucosa (infected *C. l. pidschian*) | 3.29 | | **0.002** | | -0.726 | | -0.315 | | -1.670 | | -0.121 | | 0.20 | | **0.000** | |
| Anterior content (infected *C. l. pidschian*) | vs. | Anterior mucosa (infected *C. l. pidschian*) | 3.84 | | **0.000** | | 2.186 | | -0.049 | | 2.064 | | -0.066 | | 0.16 | | **0.000** | |
| Posterior content (infected *C. l. pidschian*) | vs. | Posterior mucosa (infected *C. l. pidschian*) | 3.44 | | **0.001** | | 2.998 | | **0.010** | | 2.998 | | **0.014** | | 0.23 | | **0.002** | |
| Between anterior and posterior intestine | | | | | | | | | | | | | | | | | | |
| Anterior content (*C. l. pravdinellus*) |  | Posterior content (*C. l. pravdinellus*) | -3.02 | | **0.004** | | -0.981 | | -0.245 | | -0.211 | | -0.455 | | 0.09 | | 0.054 | |
| Anterior mucosa (*C. l. pravdinellus*) | vs. | Posterior mucosa (*C. l. pravdinellus*) | -1.42 | | 0.126 | | -2.815 | | **0.015** | | -2.668 | | **0.024** | | 0.25 | | **0.000** | |
| Washout from anterior mucosa (*C. l. pravdinellus*) | vs. | Washout from posterior mucosa (*C. l. pravdinellus*) | -2.43 | | **0.019** | | -1.435 | | -0.151 | | -1.113 | | -0.235 | | 0.13 | | **0.000** | |
| Anterior content (uninfected *C. l. pidschian*) | vs. | Posterior content (uninfected *C. l. pidschian*) | 0.29 | | 0.415 | | 1.241 | | -0.194 | | 1.521 | | -0.149 | | 0.02 | | 0.935 | |
| Anterior mucosa (uninfected *C. l. pidschian*) | vs. | Posterior mucosa (uninfected *C. l. pidschian*) | 1.75 | | 0.075 | | 1.588 | | -0.123 | | 1.214 | | -0.208 | | 0.06 | | 0.248 | |
| Washout from anterior mucosa (uninfected *C. l. pidschian*) | vs. | Washout from posterior mucosa (uninfected *C. l. pidschian*) | -0.08 | | 0.478 | | -0.952 | | -0.254 | | -1.217 | | -0.209 | | 0.08 | | 0.176 | |
| Anterior content (infected *C. l. pidschian*) | vs. | Posterior content (infected *C. l. pidschian*) | -0.34 | | 0.399 | | -1.154 | | -0.212 | | -0.879 | | -0.288 | | 0.07 | | 0.337 | |
| Anterior mucosa (infected *C. l. pidschian*) | vs. | Posterior mucosa (infected *C. l. pidschian*) | 0.22 | | 0.437 | | 0.408 | | -0.401 | | 0.725 | | -0.324 | | 0.08 | | 0.457 | |
| Washout from anterior mucosa (infected *C. l. pidschian*) | vs. | Washout from posterior mucosa (infected *C. l. pidschian*) | -1.88 | | 0.059 | | -0.344 | | -0.416 | | 0.432 | | -0.395 | | 0.25 | | 0.066 | |
| Between mucosa and their washout | | | | | | | | | | | | | | | | | | |
| Stomach mucosa (*C. l. pravdinellus*) | vs. | Washout from stomach mucosa (*C. l. pravdinellus*) | -2.03 | | **0.045** | | -2.311 | | -0.039 | | -1.800 | | -0.103 | | 0.04 | | 0.349 | |
| Anterior mucosa (*C. l. pravdinellus*) | vs. | Washout from anterior mucosa (*C. l. pravdinellus*) | -0.16 | | 0.452 | | -3.069 | | **0.008** | | -3.468 | | **0.005** | | 0.22 | | **0.001** | |
| Posterior mucosa (*C. l. pravdinellus*) | vs. | Washout from posterior mucosa (*C. l. pravdinellus*) | -1.23 | | 0.165 | | -0.401 | | -0.403 | | -0.951 | | -0.269 | | 0.17 | | **0.000** | |
| Stomach mucosa (uninfected *C. l. pidschian*) | vs. | Washout from stomach mucosa (uninfected *C. l. pidschian*) | 2.08 | | **0.042** | | -0.532 | | -0.365 | | 0.008 | | -0.497 | | 0.10 | | **0.011** | |
| Anterior mucosa (uninfected *C. l. pidschian*) | vs. | Washout from anterior mucosa (uninfected *C. l. pidschian*) | 2.02 | | **0.047** | | 2.252 | | -0.045 | | 2.237 | | -0.053 | | 0.27 | | **0.001** | |
| Posterior mucosa (uninfected *C. l. pidschian*) | vs. | Washout from posterior mucosa (uninfected *C. l. pidschian*) | 0.26 | | 0.426 | | -0.306 | | -0.424 | | -0.221 | | -0.453 | | 0.16 | | **0.012** | |
| Stomach mucosa (infected *C. l. pidschian*) | vs. | Washout from stomach mucosa (infected *C. l. pidschian*) | -0.81 | | 0.264 | | 0.434 | | -0.397 | | 0.922 | | -0.278 | | 0.18 | | **0.000** | |
| Anterior mucosa (infected *C. l. pidschian*) | vs. | Washout from anterior mucosa (infected *C. l. pidschian*) | 1.60 | | 0.096 | | 0.664 | | -0.330 | | 0.099 | | -0.474 | | 0.12 | | 0.125 | |
| Posterior mucosa (infected *C. l. pidschian*) | vs. | Washout from posterior mucosa (infected *C. l. pidschian*) | -0.60 | | 0.327 | | -0.105 | | -0.474 | | -0.151 | | -0.464 | | 0.09 | | 0.498 | |
| Between stomach and intestine | | | | | | | | | | | | | | | | | | |
| Anterior content (*C. l. pravdinellus*) | vs. | Stomach content (*C. l. pravdinellus*) | -2.38 | | **0.021** | | -1.655 | | -0.115 | | -0.876 | | -0.288 | | 0.19 | | **0.001** | |
| Anterior mucosa (*C. l. pravdinellus*) | vs. | Stomach mucosa (*C. l. pravdinellus*) | -1.88 | | 0.060 | | -2.250 | | -0.045 | | -2.127 | | -0.062 | | 0.28 | | **0.000** | |
| Posterior content (*C. l. pravdinellus*) | vs. | Stomach content (*C. l. pravdinellus*) | 0.36 | | 0.393 | | -0.799 | | -0.289 | | -0.713 | | -0.327 | | 0.16 | | **0.004** | |
| Posterior mucosa (*C. l. pravdinellus*) | vs. | Stomach mucosa (*C. l. pravdinellus*) | -0.43 | | 0.377 | | 0.639 | | -0.336 | | 0.611 | | -0.351 | | 0.17 | | **0.001** | |
| Anterior content (uninfected *C. l. pidschian*) | vs. | Stomach content (uninfected *C. l. pidschian*) | 0.02 | | 0.492 | | 1.119 | | -0.214 | | 2.097 | | -0.064 | | 0.08 | | **0.003** | |
| Anterior mucosa (uninfected *C. l. pidschian*) | vs. | Stomach mucosa (uninfected *C. l. pidschian*) | -1.81 | | 0.067 | | -0.352 | | -0.414 | | -0.469 | | -0.391 | | 0.09 | | **0.009** | |
| Posterior content (uninfected *C. l. pidschian*) | vs. | Stomach content (uninfected *C. l. pidschian*) | -0.56 | | 0.341 | | -0.077 | | -0.480 | | 0.645 | | -0.344 | | 0.06 | | 0.079 | |
| Posterior mucosa (uninfected *C. l. pidschian*) | vs. | Stomach mucosa (uninfected *C. l. pidschian*) | -0.85 | | 0.258 | | -2.010 | | -0.065 | | -1.737 | | -0.111 | | 0.08 | | **0.044** | |
| Anterior content (infected *C. l. pidschian*) | vs. | Stomach content (infected *C. l. pidschian*) | -1.85 | | 0.063 | | 0.549 | | -0.361 | | 1.347 | | -0.182 | | 0.07 | | 0.248 | |
| Anterior mucosa (infected *C. l. pidschian*) | vs. | Stomach mucosa (infected *C. l. pidschian*) | -1.68 | | 0.084 | | -2.318 | | -0.039 | | -2.374 | | -0.040 | | 0.08 | | 0.189 | |
| Posterior content (infected *C. l. pidschian*) | vs. | Stomach content (infected *C. l. pidschian*) | -0.08 | | 0.478 | | 1.655 | | -0.116 | | 2.162 | | -0.060 | | 0.18 | | **0.000** | |
| Posterior mucosa (infected *C. l. pidschian*) | vs. | Stomach mucosa (infected *C. l. pidschian*) | 0.25 | | 0.428 | | -2.239 | | -0.045 | | -2.596 | | -0.029 | | 0.13 | | **0.056** | |

Table S4. Test effect of factor «Fish» on microbial community of cestodes parasitizing the intestine of *C. l. pidschian* using the ADONIS test on Bray-Curtis matrix. The bold character indicates significance at p<0.05.

| Factor | Df | Sum of Sqs | R2 | F | Pr(>F) |
| --- | --- | --- | --- | --- | --- |
| Cestodes | 7 | 5,59 | 0,11255 | 10,1635 | **1,00E-04** |
| Fish | 8 | 26,258 | 0,52865 | 41,7707 | **1,00E-04** |
| Cestodes:Fish | 53 | 11,224 | 0,22591 | 2,6944 | **1,00E-04** |
| Residual | 84 | - | - | 6,601 | 1,33E-01 |
| Total | 152 | - | - | 49,67 | 1,00E+00 |

Table S5. Significantly different biomarkers in relative abundance of microbial community among different segments of the digestive tract of *C. l. pidschian* and washout of cestodes using the Linear discriminant analysis effect size (LEfSe) algorithm. A p-value of < 0.01 and LDA score ≥ 4.0 were considered significant. AC – Anterior content, AM – Anterior mucosa, PC – Posterior content, PM – Posterior mucosa.

| **Phylum** | **Class** | **Order** | **Family** | **Genus** | **Type** | **logarithmic LDA score** | **p-value** |
| --- | --- | --- | --- | --- | --- | --- | --- |
| Proteobacteria | Alphaproteobacteria | Rhizobiales | *Rhizobiaceae* | NA | D0 | 4.87 | 5.80E-09 |
| Proteobacteria | Alphaproteobacteria | Sphingomonadales | *Sphingomonadaceae* | *Sphingobium* | D1 | 4.95 | 5.10E-03 |
| Proteobacteria | Alphaproteobacteria | Sphingomonadales | *Sphingomonadaceae* |  | D1 | 4.96 | 5.50E-03 |
| Proteobacteria | Alphaproteobacteria | Sphingomonadales |  |  | D1 | 4.96 | 5.50E-03 |
| Firmicutes | Bacilli | Mycoplasmatales | *Mycoplasmataceae* | *Mycoplasma* | D2 | 5.31 | 2.80E-03 |
| Firmicutes | Bacilli | Mycoplasmatales | *Mycoplasmataceae* |  | D2 | 5.31 | 2.80E-03 |
| Firmicutes | Bacilli | Mycoplasmatales |  |  | D2 | 5.31 | 2.80E-03 |
| Firmicutes | Bacilli |  |  |  | D2 | 5.31 | 4.40E-03 |
| Proteobacteria | Alphaproteobacteria | Azospirillales | *Azospirillaceae* | *Azospirillum* | D3 | 4.52 | 4.70E-07 |
| Proteobacteria | Alphaproteobacteria | Azospirillales | *Azospirillaceae* |  | D3 | 4.52 | 4.70E-07 |
| Proteobacteria | Alphaproteobacteria | Azospirillales |  |  | D3 | 4.52 | 4.70E-07 |
| Proteobacteria | Gammaproteobacteria | Enterobacterales | *Erwiniaceae* | NA | D4 | 4.67 | 2.20E-11 |
| Proteobacteria | Gammaproteobacteria | Enterobacterales | *Erwiniaceae* |  | D4 | 4.67 | 2.20E-11 |
| Proteobacteria | Gammaproteobacteria | Enterobacterales |  |  | D4 | 4.70 | 5.40E-05 |
| Actinobacteriota | Actinobacteria | Micrococcales |  |  | D5 | 4.04 | 1.40E-04 |
| Proteobacteria | Alphaproteobacteria | Rhizobiales | *Rhizobiaceae* | *Allorhizobium-Neorhizobium-Pararhizobium-Rhizobium* | D6 | 4.75 | 4.10E-10 |
| Proteobacteria | Alphaproteobacteria | Rhizobiales | *Rhizobiaceae* |  | D6 | 4.87 | 1.30E-05 |
| Proteobacteria | Gammaproteobacteria | Pseudomonadales | *Moraxellaceae* | *Acinetobacter* | D6 | 5.10 | 1.40E-07 |
| Proteobacteria | Gammaproteobacteria | Pseudomonadales | *Moraxellaceae* |  | D6 | 5.10 | 3.20E-07 |
| Deinococcota | Deinococci | Thermales | *Thermaceae* | *Thermus* | D7 | 4.31 | 9.00E-08 |
| Deinococcota | Deinococci | Thermales | *Thermaceae* |  | D7 | 4.32 | 8.70E-08 |
| Deinococcota | Deinococci | Thermales |  |  | D7 | 4.32 | 8.70E-08 |
| Deinococcota | Deinococci |  |  |  | D7 | 4.32 | 7.60E-08 |
| Deinococcota |  |  |  |  | D7 | 4.32 | 7.60E-08 |
| Proteobacteria | Gammaproteobacteria | Burkholderiales | *Comamonadaceae* | *Comamonas* | D7 | 4.92 | 2.90E-08 |
| Proteobacteria | Gammaproteobacteria | Burkholderiales | *Burkholderiaceae* | *Cupriavidus* | D7 | 4.02 | 8.00E-07 |
| Proteobacteria | Gammaproteobacteria | Burkholderiales | *Comamonadaceae* | NA | D7 | 4.84 | 3.60E-04 |
| Proteobacteria | Gammaproteobacteria | Burkholderiales | *Burkholderiaceae* |  | D7 | 4.04 | 1.60E-06 |
| Proteobacteria | Gammaproteobacteria | Burkholderiales | *Comamonadaceae* |  | D7 | 5.23 | 1.60E-05 |
| Proteobacteria | Gammaproteobacteria | Burkholderiales |  |  | D7 | 5.24 | 1.70E-04 |
| Proteobacteria | Gammaproteobacteria | Xanthomonadales | *Xanthomonadaceae* | *Stenotrophomonas* | D7 | 4.35 | 1.30E-10 |
| Proteobacteria | Gammaproteobacteria | Xanthomonadales | *Xanthomonadaceae* |  | D7 | 4.35 | 1.60E-13 |
| Proteobacteria | Gammaproteobacteria | Xanthomonadales |  |  | D7 | 4.35 | 3.00E-13 |
| Actinobacteriota | Actinobacteria |  |  |  | AC | 4.15 | 6.50E-04 |
| Actinobacteriota |  |  |  |  | AC | 4.44 | 8.50E-06 |
| Bacteroidota | Bacteroidia | Bacteroidales | NA | NA | AC | 4.30 | 2.00E-09 |
| Bacteroidota | Bacteroidia | Bacteroidales | NA |  | AC | 4.30 | 2.00E-09 |
| Bacteroidota | Bacteroidia | Bacteroidales |  |  | AC | 4.30 | 1.50E-10 |
| Bacteroidota | Bacteroidia |  |  |  | AC | 4.30 | 8.00E-09 |
| Bacteroidota |  |  |  |  | AC | 4.31 | 6.30E-09 |
| Cyanobacteria | Cyanobacteriia | Chloroplast | NA | NA | AC | 4.94 | 2.00E-17 |
| Cyanobacteria | Cyanobacteriia | Chloroplast | NA |  | AC | 4.94 | 2.00E-17 |
| Cyanobacteria | Cyanobacteriia | Chloroplast |  |  | AC | 4.94 | 2.00E-17 |
| Cyanobacteria | Cyanobacteriia |  |  |  | AC | 5.04 | 9.40E-19 |
| Cyanobacteria |  |  |  |  | AC | 5.04 | 9.40E-19 |
| Proteobacteria | Gammaproteobacteria | Burkholderiales | SC_I_84 | NA | AC | 4.09 | 1.20E-23 |
| Proteobacteria | Gammaproteobacteria | Burkholderiales | SC_I_84 |  | AC | 4.09 | 1.20E-23 |
| Verrucomicrobiota | Verrucomicrobiae | Verrucomicrobiales | *Rubritaleaceae* | *Luteolibacter* | AC | 4.55 | 1.40E-22 |
| Verrucomicrobiota | Verrucomicrobiae | Verrucomicrobiales | *Rubritaleaceae* |  | AC | 4.55 | 1.40E-22 |
| Verrucomicrobiota | Verrucomicrobiae | Verrucomicrobiales |  |  | AC | 4.56 | 1.40E-22 |
| Cyanobacteria | Cyanobacteriia | Synechococcales | *Cyanobiaceae* | *Cyanobium* | PC | 4.16 | 4.60E-19 |
| Cyanobacteria | Cyanobacteriia | Synechococcales | *Cyanobiaceae* |  | PC | 4.20 | 4.90E-19 |
| Cyanobacteria | Cyanobacteriia | Synechococcales |  |  | PC | 4.20 | 4.90E-19 |
| Planctomycetota | Planctomycetes | Pirellulales | *Pirellulaceae* |  | PC | 4.28 | 1.50E-23 |
| Planctomycetota | Planctomycetes | Pirellulales |  |  | PC | 4.28 | 1.50E-23 |
| Planctomycetota | Planctomycetes |  |  |  | PC | 4.58 | 2.90E-23 |
| Planctomycetota |  |  |  |  | PC | 4.58 | 2.80E-23 |
| Proteobacteria | Alphaproteobacteria | Rhizobiales | *Beijerinckiaceae* | NA | PC | 4.16 | 1.20E-16 |
| Proteobacteria | Alphaproteobacteria | Rhizobiales | *Beijerinckiaceae* |  | PC | 4.21 | 3.10E-04 |
| Proteobacteria | Alphaproteobacteria | Rhizobiales | *Hyphomicrobiaceae* |  | PC | 4.10 | 2.40E-22 |
| Proteobacteria | Alphaproteobacteria | Rhizobiales | NA | NA | PC | 4.37 | 1.90E-25 |
| Proteobacteria | Alphaproteobacteria | Rhizobiales | NA |  | PC | 4.37 | 1.90E-25 |
| Proteobacteria | Alphaproteobacteria | Rhizobiales | *Xanthobacteraceae* | NA | PC | 4.21 | 6.80E-17 |
| Proteobacteria | Alphaproteobacteria | Rhizobiales | *Xanthobacteraceae* |  | PC | 4.29 | 2.20E-16 |
| Proteobacteria | Alphaproteobacteria | Rhizobiales |  |  | PC | 4.99 | 3.20E-08 |
| Proteobacteria | Alphaproteobacteria | Rhodobacterales | *Rhodobacteraceae* | NA | PC | 4.59 | 6.60E-21 |
| Proteobacteria | Alphaproteobacteria | Rhodobacterales | *Rhodobacteraceae* |  | PC | 4.66 | 6.00E-15 |
| Proteobacteria | Alphaproteobacteria | Rhodobacterales |  |  | PC | 4.66 | 6.00E-15 |
| Proteobacteria | Alphaproteobacteria |  |  |  | PC | 5.19 | 2.10E-03 |
| Proteobacteria | Gammaproteobacteria | Methylococcales |  |  | PC | 4.05 | 3.50E-25 |
| Proteobacteria | Gammaproteobacteria | NA | NA | NA | PC | 4.13 | 1.00E-21 |
| Proteobacteria | Gammaproteobacteria | NA | NA |  | PC | 4.13 | 1.00E-21 |
| Proteobacteria | Gammaproteobacteria | NA |  |  | PC | 4.13 | 1.00E-21 |
| Verrucomicrobiota | Verrucomicrobiae | Chthoniobacterales | *Chthoniobacteraceae* | Candidatus *Udaeobacter* | PC | 4.02 | 5.10E-25 |
| Verrucomicrobiota | Verrucomicrobiae | Chthoniobacterales | *Chthoniobacteraceae* |  | PC | 4.28 | 2.40E-23 |
| Verrucomicrobiota | Verrucomicrobiae | Chthoniobacterales |  |  | PC | 4.35 | 3.60E-22 |
| Verrucomicrobiota | Verrucomicrobiae |  |  |  | PC | 4.75 | 1.20E-22 |
| Verrucomicrobiota |  |  |  |  | PC | 4.78 | 1.70E-22 |
| Firmicutes | Bacilli | Bacillales | *Bacillaceae* |  | AM | 4.01 | 4.10E-05 |
| Firmicutes | Bacilli | Bacillales |  |  | AM | 4.02 | 5.10E-05 |
| Firmicutes | Clostridia | NA | NA | NA | PM | 4.91 | 2.30E-05 |
| Firmicutes | Clostridia | NA | NA |  | PM | 4.90 | 2.30E-05 |
| Firmicutes | Clostridia | NA |  |  | PM | 4.91 | 1.10E-12 |
| Firmicutes | Clostridia |  |  |  | PM | 4.93 | 2.20E-08 |
| Proteobacteria | Gammaproteobacteria | Pseudomonadales | *Pseudomonadaceae* | *Pseudomonas* | PM | 5.38 | 6.90E-07 |
| Proteobacteria | Gammaproteobacteria | Pseudomonadales | *Pseudomonadaceae* |  | PM | 5.40 | 6.90E-07 |
| Proteobacteria | Gammaproteobacteria | Pseudomonadales |  |  | PM | 5.41 | 1.30E-06 |
| Proteobacteria | Gammaproteobacteria |  |  |  | PM | 5.36 | 6.70E-03 |
